# Bright Side of *Fusarium oxysporum*: Secondary Metabolites Bioactivities and Industrial Relevance in Biotechnology and Nanotechnology

**DOI:** 10.3390/jof7110943

**Published:** 2021-11-08

**Authors:** Sabrin R. M. Ibrahim, Alaa Sirwi, Basma G. Eid, Shaimaa G. A. Mohamed, Gamal A. Mohamed

**Affiliations:** 1 Preparatory Year Program, Batterjee Medical College, Jeddah 21442, Saudi Arabia; 2 Department of Pharmacognosy, Faculty of Pharmacy, Assiut University, Assiut 71526, Egypt; 3 Department of Natural Products and Alternative Medicine, Faculty of Pharmacy, King Abdulaziz University, Jeddah 21589, Saudi Arabia; asirwi@kau.edu.sa (A.S.); gahussein@kau.edu.sa (G.A.M.); 4 Department of Pharmacology and Toxicology, Faculty of Pharmacy, King Abdulaziz University, Jeddah 21589, Saudi Arabia; beid@kau.edu.sa; 5 Faculty of Dentistry, British University, Suez Desert Road, Cairo 11837, Egypt; shaimaag1973@gmail.com; 6 Department of Pharmacognosy, Faculty of Pharmacy, Al-Azhar University, Assiut Branch, Assiut 71524, Egypt

**Keywords:** *Fusarium oxysporum*, fungi, metabolites, bioactivities, nanotechnology, industrial relevance

## Abstract

Fungi have been assured to be one of the wealthiest pools of bio-metabolites with remarkable potential for discovering new drugs. The pathogenic fungi, *Fusarium oxysporum* affects many valuable trees and crops all over the world, producing wilt. This fungus is a source of different enzymes that have variable industrial and biotechnological applications. Additionally, it is widely employed for the synthesis of different types of metal nanoparticles with various biotechnological, pharmaceutical, industrial, and medicinal applications. Moreover, it possesses a mysterious capacity to produce a wide array of metabolites with a broad spectrum of bioactivities such as alkaloids, jasmonates, anthranilates, cyclic peptides, cyclic depsipeptides, xanthones, quinones, and terpenoids. Therefore, this review will cover the previously reported data on *F. oxysporum*, especially its metabolites and their bioactivities, as well as industrial relevance in biotechnology and nanotechnology in the period from 1967 to 2021. In this work, 180 metabolites have been listed and 203 references have been cited.

## 1. Introduction

**Scheme 1:**
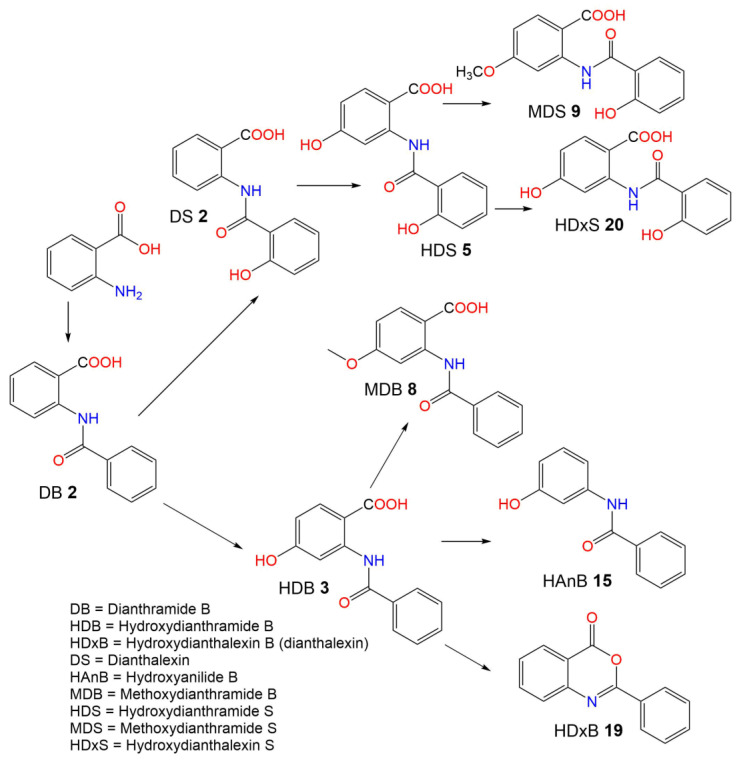
Possible biosynthetic pathway for the formation of anthranilic acid derivatives [56,182,183].

**Figure 1:**
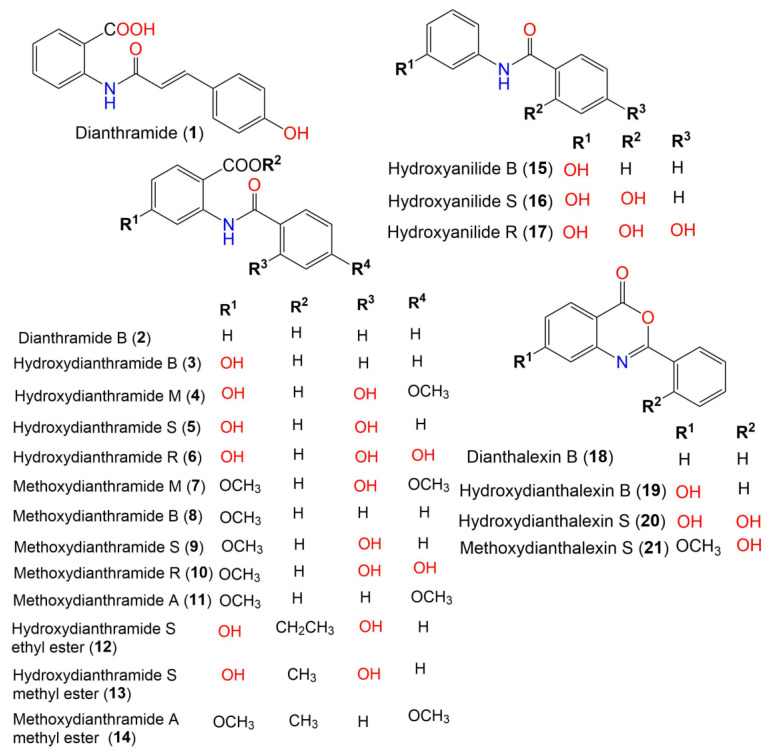
Structures of anthranilic acid derivatives (**1**–**21**) isolated from *F. oxysporum*.

**Figure 2:**
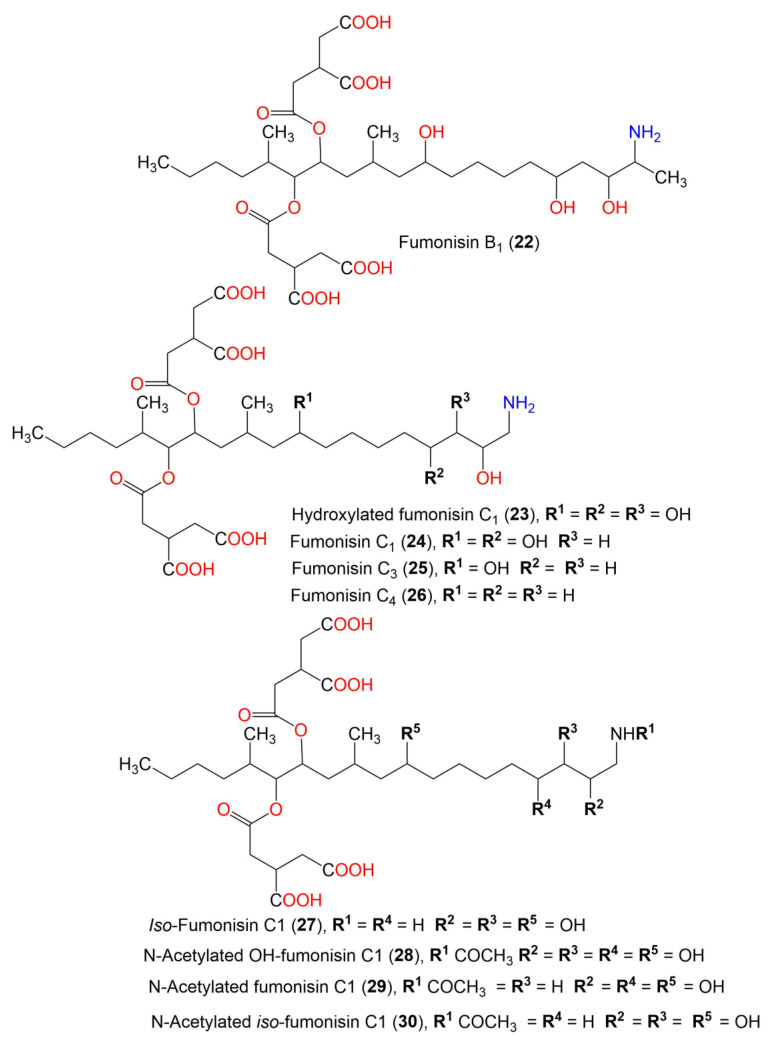
Structures of fumonisins **22**–**30** isolated from *F. oxysporum*.

**Figure 3:**
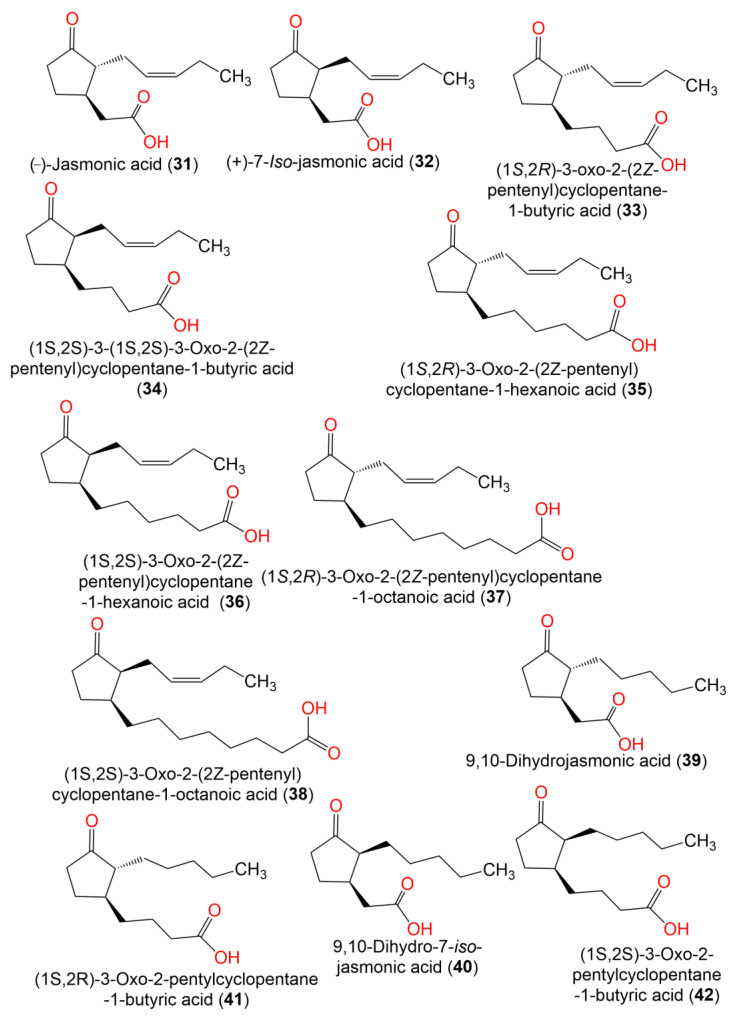
Structures of jasmonates derivatives (**31**–**42**) isolated from *F. oxysporum*.

**Figure 4:**
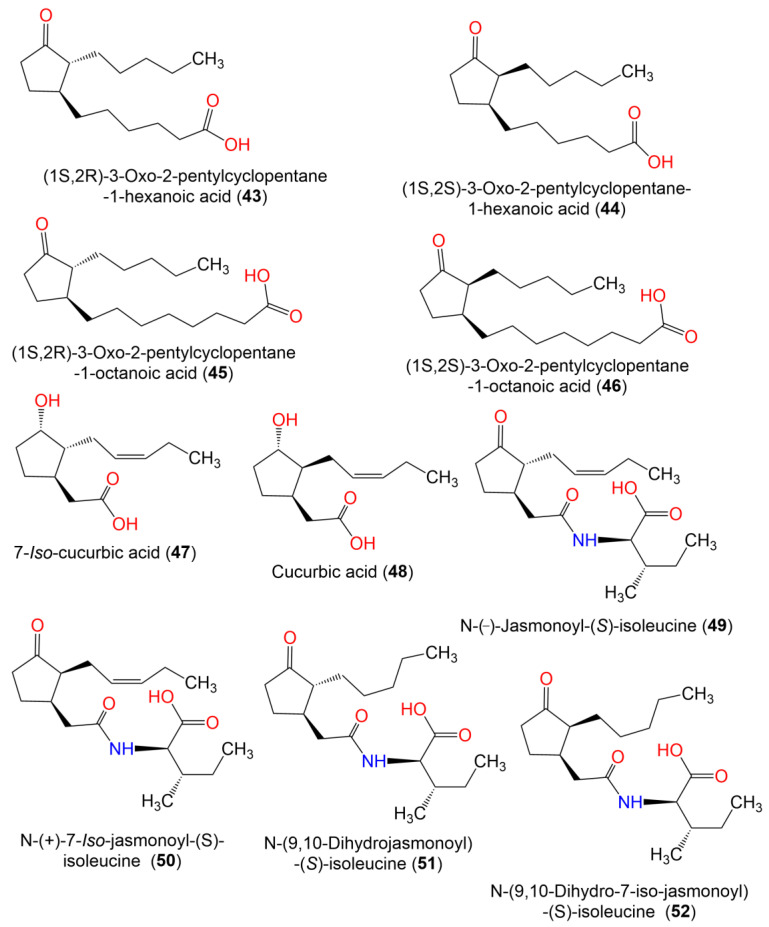
Structures of jasmonates derivatives (**43**–**52**) isolated from *F. oxysporum*.

**Figure 5:**
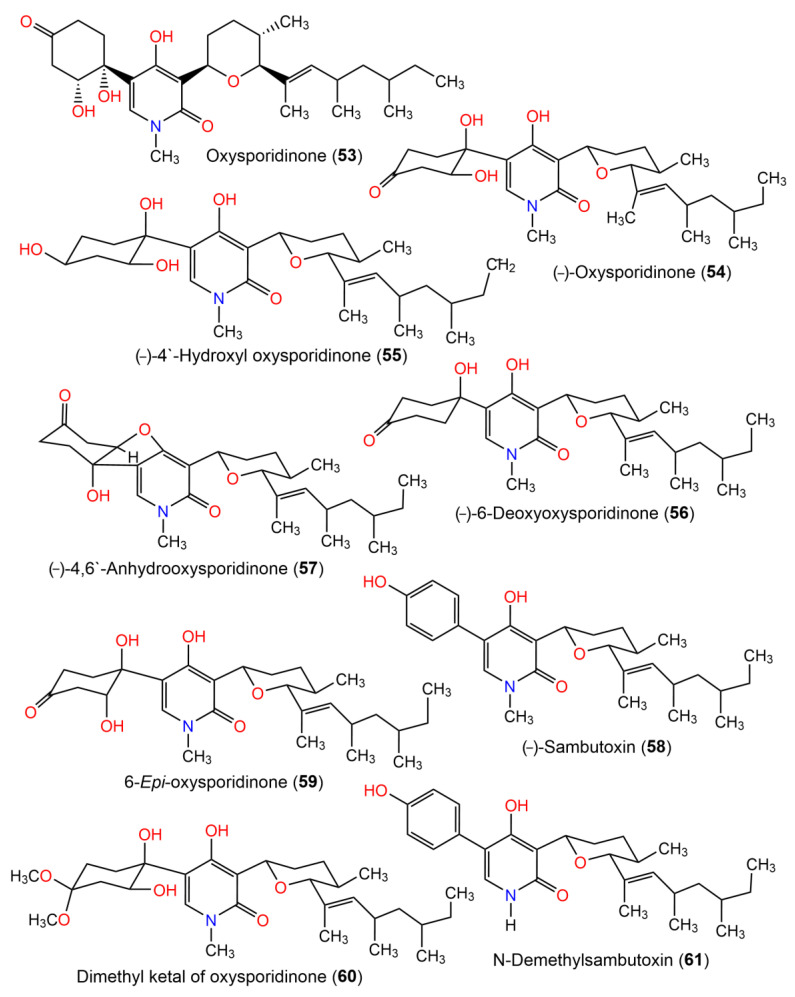
Structures of pyridinone alkaloids (**53**–**61**) isolated from *F. oxysporum*.

**Scheme 2:**
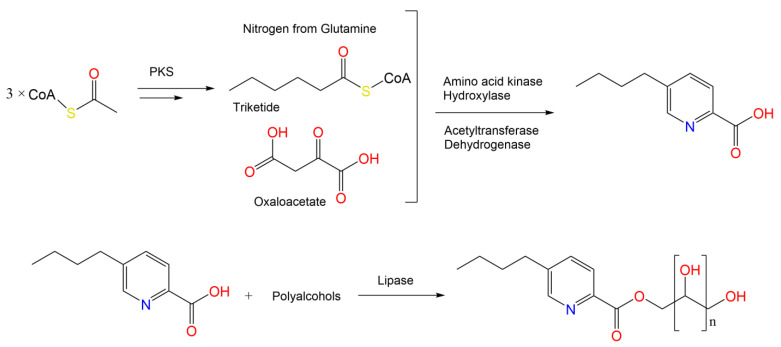
Possible biosynthetic pathway of fusaricates A-G (**65**–**71**) [69].

**Figure 6:**
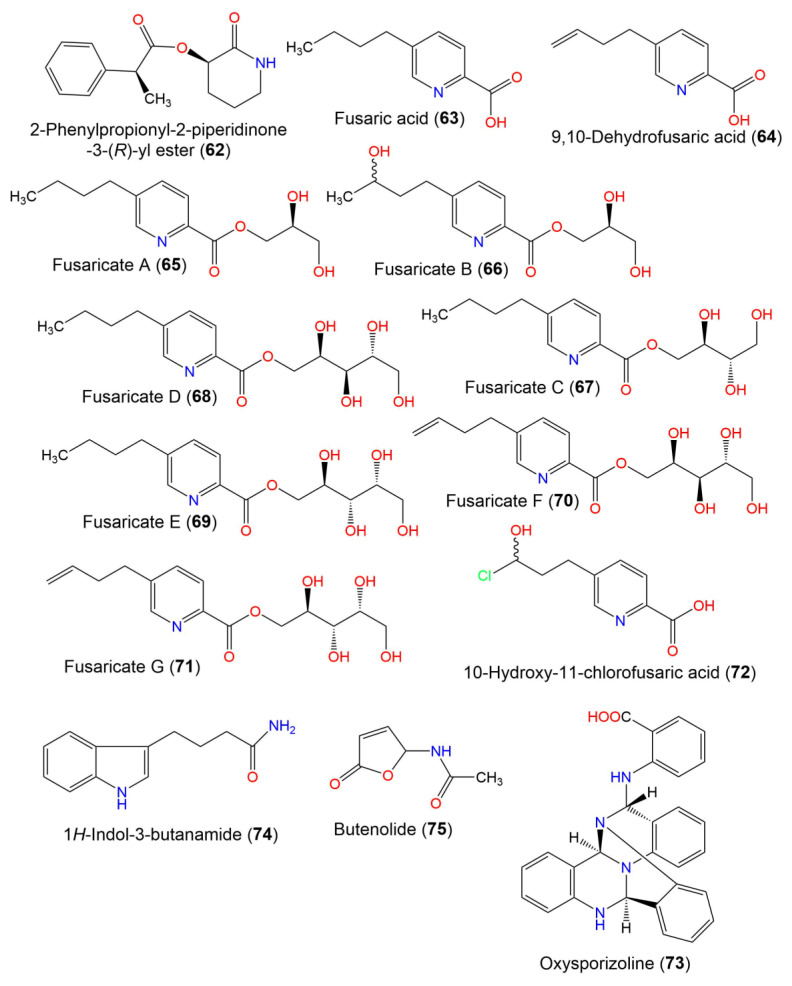
Structures of alkaloids (**62**–**75**) isolated from *F. oxysporum*.

**Figure 7:**
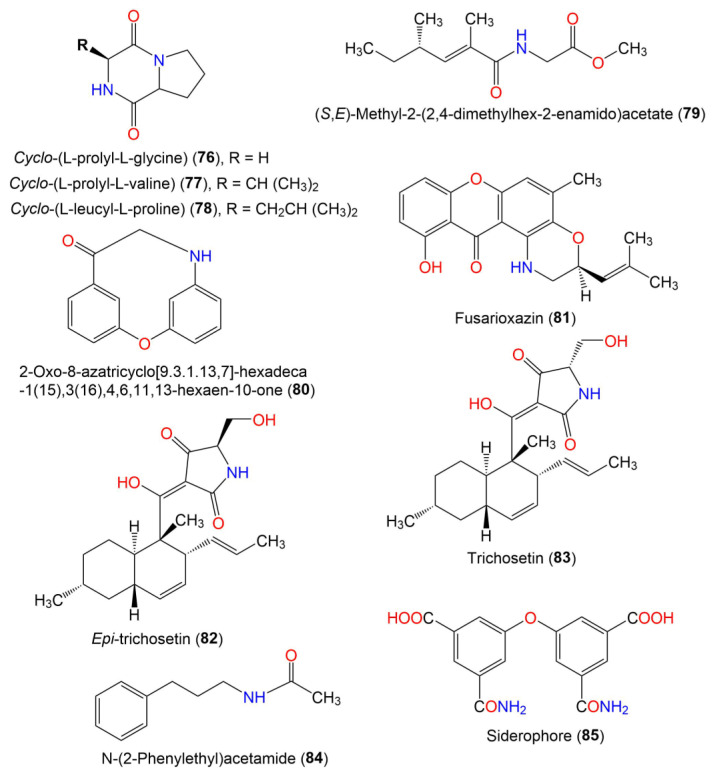
Structures of alkaloids (**76**–**85**) isolated from *F. oxysporum*.

**Figure 8:**
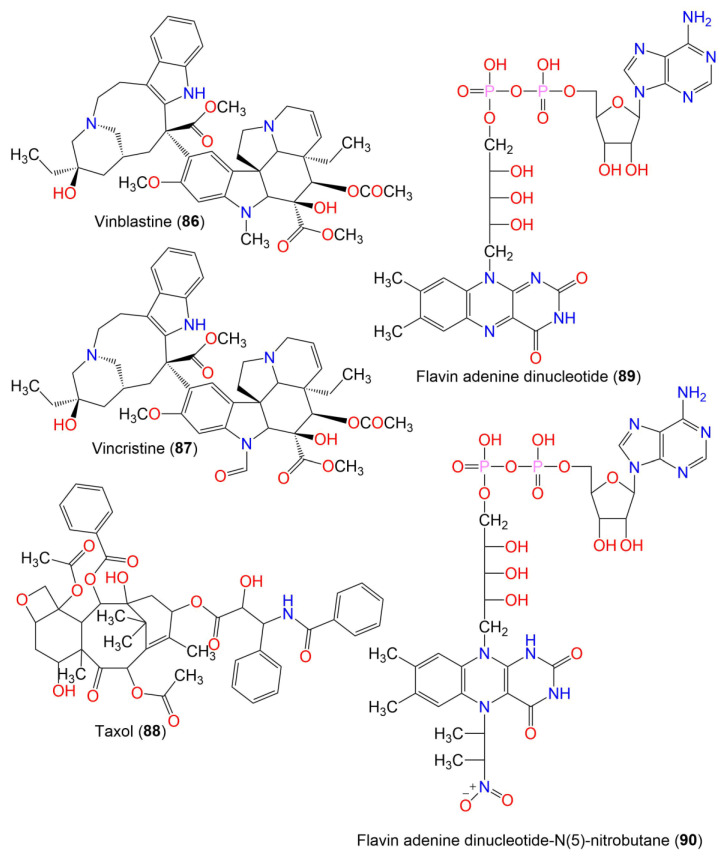
Structures of alkaloids (**86**–**90**) isolated from *F. oxysporum*.

**Figure 9:**
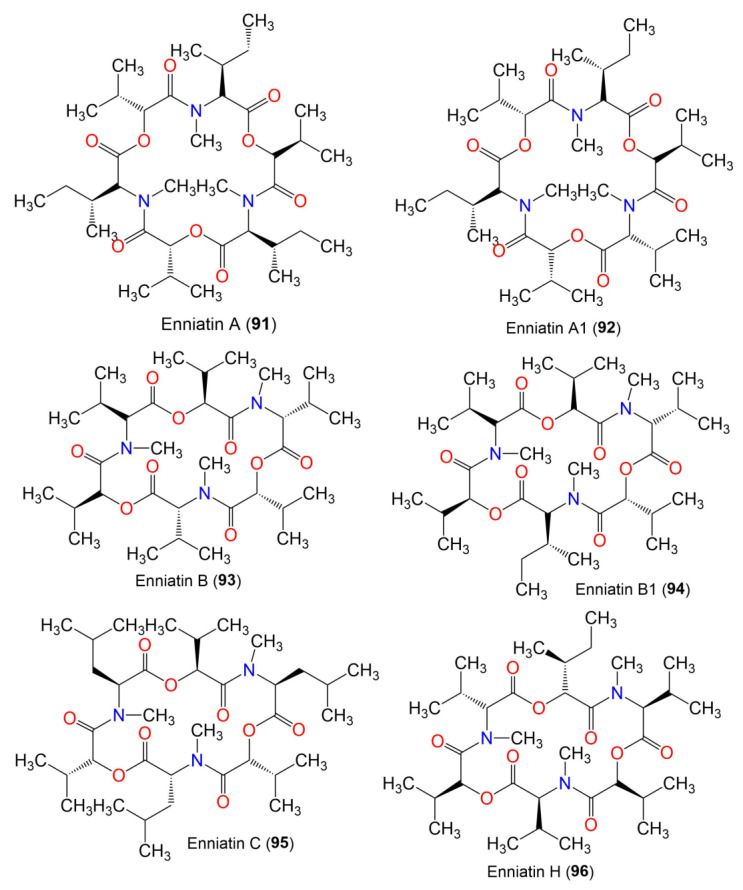
Structures of cyclic depsipeptides (**91**–**96**) isolated from *F. oxysporum*.

**Figure 10:**
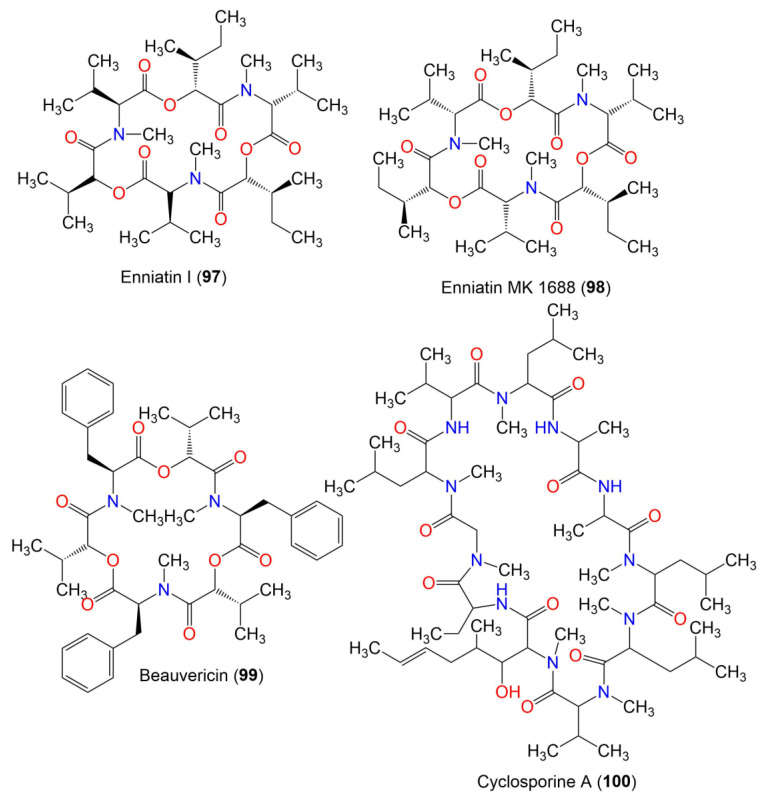
Structures of cyclic depsipeptides (**97**–**99**) and cyclic peptide (**100**) isolated from *F. oxysporum*.

**Figure 11:**
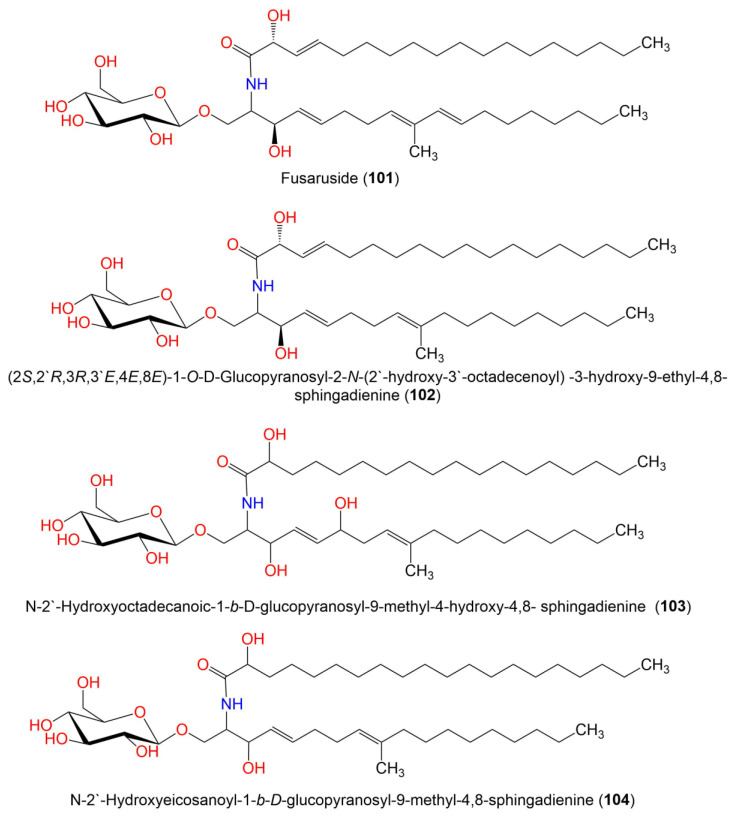
Structures of glucosylceramides (**101**–**104**) isolated from *F. oxysporum*.

**Figure 12:**
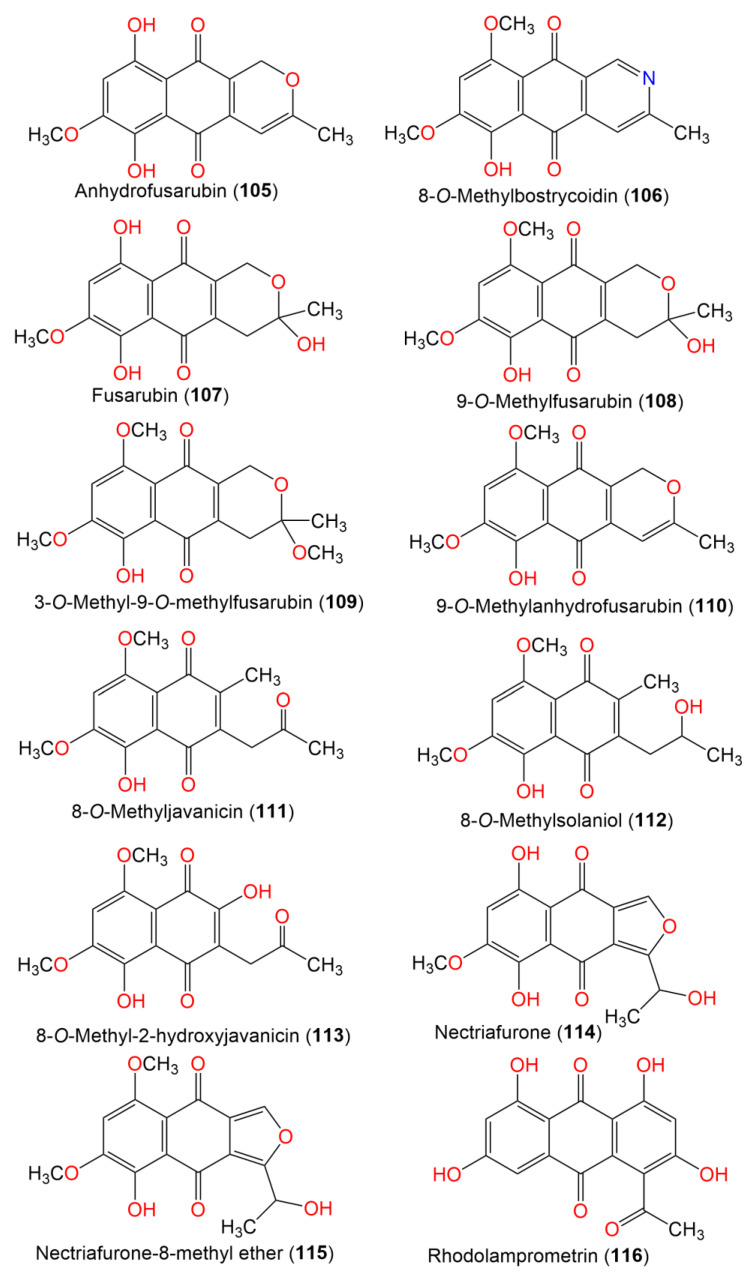
Structures of naphthoquinone (**105**–**115**) and anthraquinone (**116**) derivatives isolated from *F. oxysporum*.

**Scheme 3:**
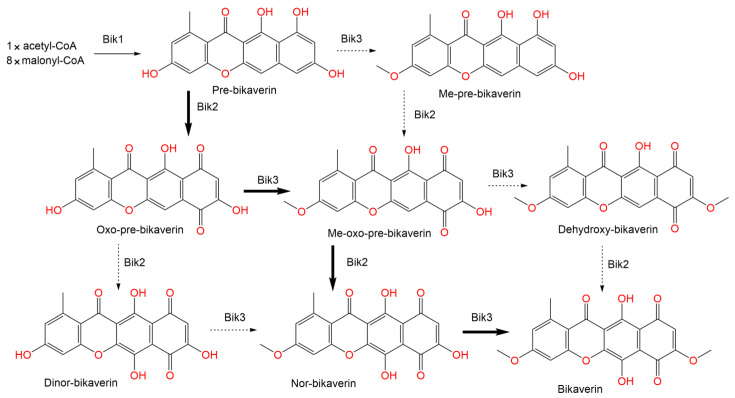
Putative biosynthetic pathway of **119** and its intermediates. The bold arrows represent the preferred pathway and dashed lines represented other possible reaction steps [196,197,198].

**Figure 13:**
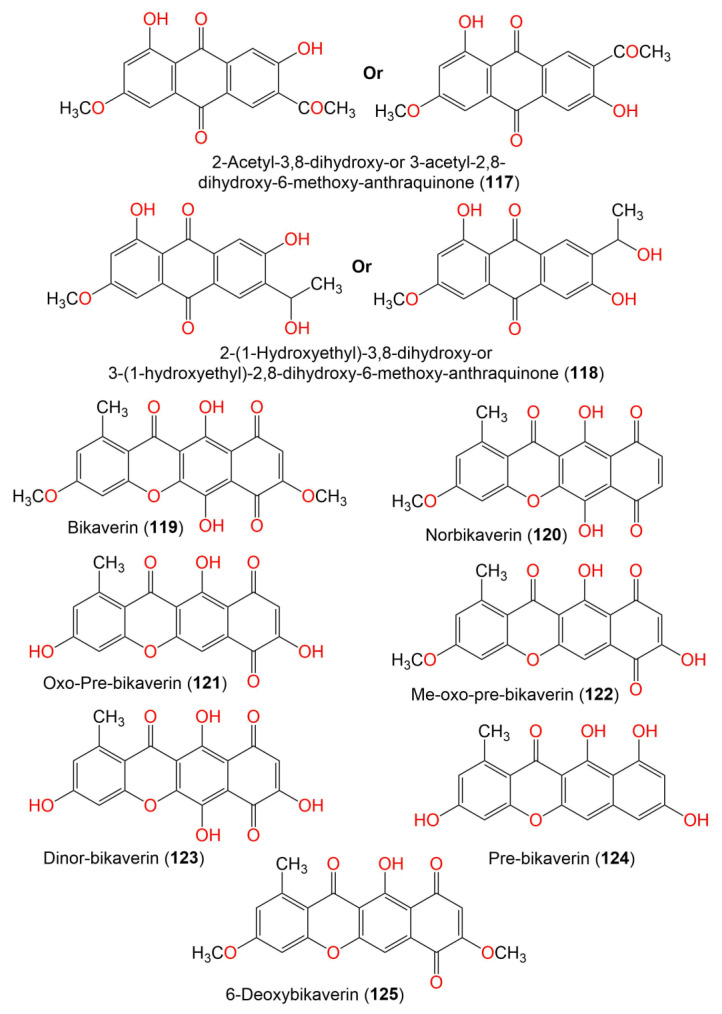
Structures of xanthone derivatives (**117**–**125**) isolated from *F. oxysporum*.

**Figure 14:**
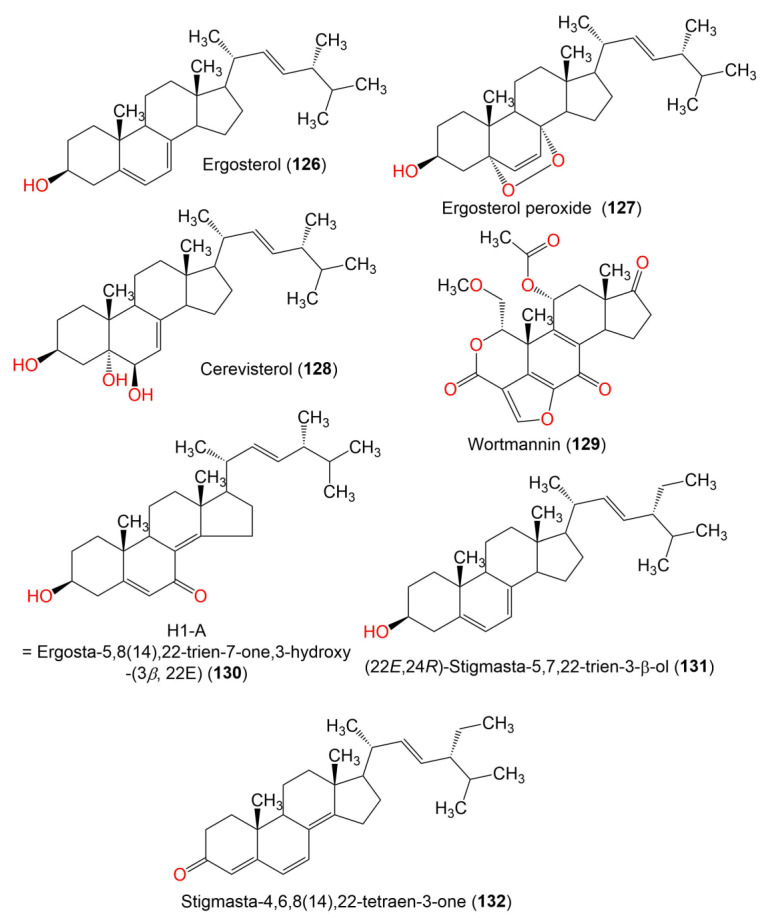
Structures of sterols (**126**–**132**) isolated from *F. oxysporum*.

**Figure 15:**
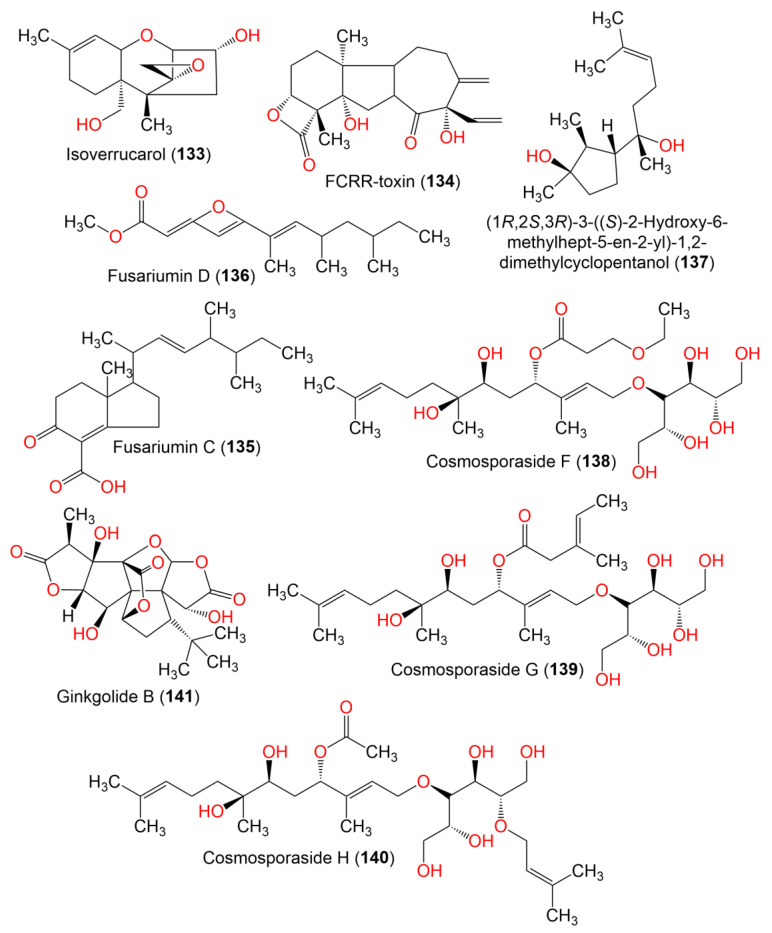
Structures of terpenoids (**133**–**141**) isolated from *F. oxysporum*.

**Figure 16:**
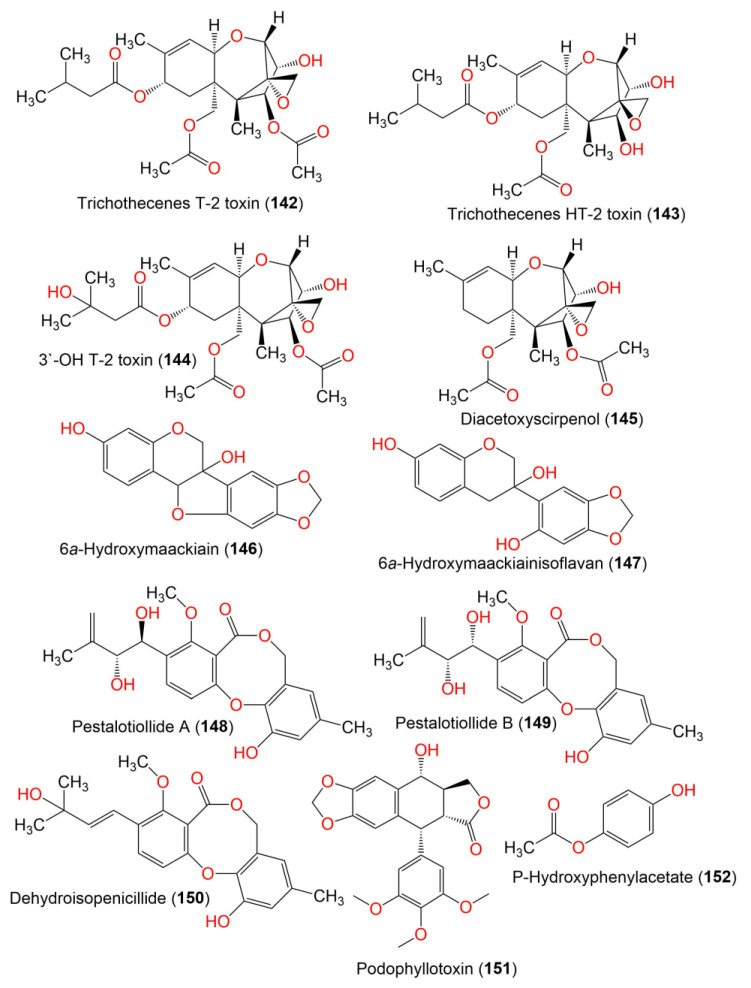
Structures of terpenoids (**142**–**145**), flavonoids (**146** and **147**), depsidones (**148**–**150**), lignan (**151**), and phenolic compound (**152**).

**Figure 17:**
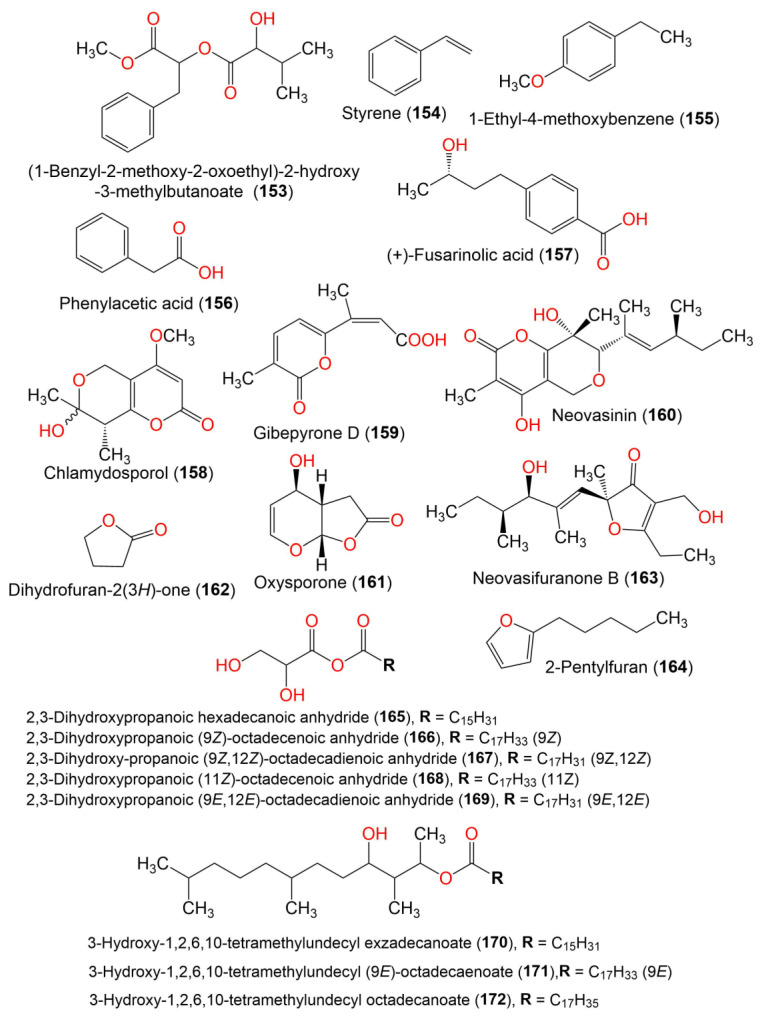
Structures of aromatic compounds (**153**–**157**), pyran (**158**–**160)** and furan **(161**–**164**) derivatives, and aliphatic acids (**165**–**172**) isolated from *F. oxysporum*.

**Figure 18:**
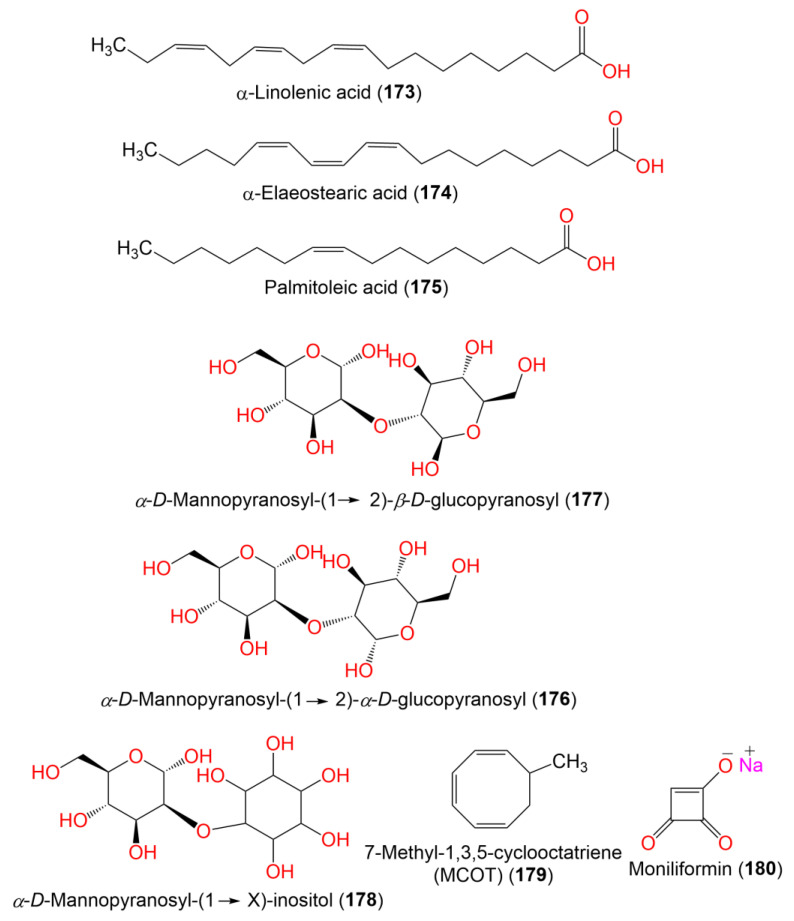
Structures of fatty acids (**173**–**175**) and sugar derivatives (176–**180**) isolated from *F. oxysporum*.

**Figure 19:**
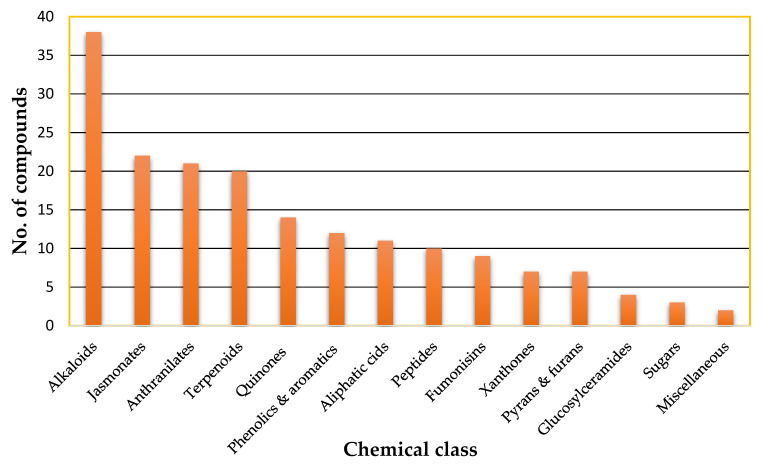
Different classes of metabolites reported from *F. oxysporum*.

Fungi are eukaryotic microorganisms that settled mostly in all kinds of environments and have fundamental roles in maintaining the environmental balance [1,2]. It has been stated that only about 5% of 2.2 to 3.8 million different fungal species on earth have been taxonomically characterized [3,4]. Fungi have been considered as one of the wealthiest pools of natural metabolites with unique structural features and biodiversity that have a remarkable role in developing new drugs [1,2,3,5,6,7,8,9,10]. Some fungal metabolites are also highly toxic such as *Aspergillus* mycotoxin and aflatoxin B1, which affect human health when occurring in food products [8,9,11]. Therefore, it is important to unravel the metabolites of fungal species to prevent health risks, as well as to identify new potential bioactive compounds. *Fusarium* is a genus of filamentous fungi that includes many mycotoxin producers, agronomically important plant pathogens, and opportunistic human pathogens [12]. Its species are a widespread cosmopolitan group of fungi that are found in various habitats such as water, soil, or associated with plants [13,14]. They commonly colonize subterranean and aerial plant parts, either as primary or secondary invaders [15]. Many *Fusarium* species are spread out pathogens on crops in temperate and semi-tropical regions that produce a variety of mycotoxins, causing a reduction in yield and quality of crops, as well as animal and human health risks [16]. On the other hand, some species have the potential capacity to produce a great number of metabolites with remarkable chemical diversity and significant bioactivities [11,17,18,19,20,21,22,23,24,25,26]. *Fusarium oxysporum* is the most encountered and economically important species of this genus. It includes pathogenic (plant, human, and animal) and non-pathogenic strains that even possess bio-control activity against fungal pests and some insects [27]. It is one of the soil-borne pathogens that causes vascular wilt on many plants, which is characterized by various symptoms, including leaf epinasty, vascular browning, progressive wilting, defoliation, stunting, and plant death [28]. Its species complex consists of several formae speciales (f. sp.) that collectively infect more than one hundred hosts, leading to serious losses in crops such as tomato, melon, banana, and cotton [29]. In humans, *F. oxysporum* causes invasive infections in immuno-compromised patients and it is commonly found in onychomycosis [30,31]. Many studies revealed that *F. oxysporum* showed a remarkable capacity to yield diverse classes of secondary metabolites such as alkaloids, jasmonates, anthranilates, cyclic peptides, cyclic depsipeptides, xanthones, quinones, and terpenoids with various activities such as phytotoxicity, antimicrobial, cytotoxicity, insecticidal, antioxidant, and antiangiogenic. Additionally, *F. oxysporum* possessed significant industrial and biotechnological values as a wealthy source of diverse enzymes with wide applications such as cutinases, nitrilases, glycoside hydrolases (e.g., fucosidase, α-galactopyranosidases, and xylanases), fructosyl amino acid oxidase, laccases, lipoxygenase, nitric oxide reductase, decarboxylases, keratinase, phospholipase B, and triosephosphate isomerase [32,33,34,35,36,37,38,39,40,41,42]. Further, *F. oxysporum* is widely employed for the synthesis of different types of metal nanoparticles that could have various biotechnological, pharmaceutical, industrial, and medicinal applications [43,44,45,46,47,48,49,50,51,52,53,54,55]. The intensive literature search revealed the lack of review articles that deal with *F. oxysporum* particularly the bright side of this economically valuable fungus. The current review summarized the published data regarding secondary metabolites reported from this fungus and their bioactivities. Additionally, the research progress on *F. oxysporum*, including industrial, biotechnological, and nanotechnological applications has been discussed. The studies that have appeared in literature from 1967 to 2021 are reported. The chemical classes, structures, molecular formulae and weights, hosts, places, and bioactivities of the reported metabolites have been listed (Figures 1–19 and Table 1 and Table 2). Moreover, the reported biosynthetic pathways of some major *F. oxysporum* metabolites have been included (Schemes 1–3). The aim of this work is to focus on the interests of biologists, chemists, and natural product researchers on the area of pharmaceutical drug leads development of the reported metabolites. Besides, the covered data of industrial relevance in biotechnology and nanotechnology have been discussed. The literature search for this work was performed through a computer search of data on Web of Knowledge, ScienceDirect, SCOPUS, Taylor & Francis, Wiley Online Library, Springer, PubMed, JACS, and Google Scholar.

## 2. Nanotechnological Applications

Nanotechnology holds promise in the medicine, agriculture, and pharmaceutical industries [112]. Natural nanostructures have gained more attention due to the wide spectrum of bioactivities and fewer animals, humans, and environmental toxicity. The microbial synthesis of nanoparticles is an approach of green chemistry that combines both nanotechnology and microbial biotechnology [113]. Metals nanoparticles are increasingly used in various biotechnological, pharmaceutical, and medicinal applications, including drug delivery, gene transfer, insect-pests management in agriculture, and bioelectronics devices fabrication, as well as antibacterial agents towards many pathogenic bacteria, including the MDR (multidrug-resistant) strains [114,115,116].

### 2.1. Metal Nanoparticles

Several studies reported the synthesis and characterization of metal nanoparticles (NPs) using *F. oxysporum*, as well as their bioactivities. Additionally, some studies dealt with optimizing the conditions for the synthesis of NPs by *F. oxysporum*, including temperature, media, pH, salt concentration, light intensity, the volume of filtrate, and biomass quantity [44,47,50,51,54,55]. Marcato et al., synthesized AgNPs (silver nanoparticles) using *F. oxysporum*. The incorporation of these NPs in cotton cloth was found to exhibit a bactericidal effect towards *S. aureus*, leading to its sterilization [50]. Ishida et al., synthesized AgNPs using *F. oxysporum* aqueous extract that showed significant antifungal potential towards *Cryptococcus* and *Candida* (MIC values ≤ 1.68 µg/mL) [51]. Moreover, it was found that the biosynthesized AgNPs by two *F. oxysporum* isolates exhibited higher antibacterial potential towards human-pathogenic bacteria; *E. coli*, *Proteus vulgaris*, *S. aureus*, and *K. pneumonia* than the used antibiotics. These AgNPs could be favorable antibacterial agents, especially towards MDR bacteria [44]. Ahmed et al., synthesized AgNPs using *F. oxysporum*, which inhibited some MDR species of *Staphylococcus* and *Enterobacteriaceae* (conc. 50% *v*/*v*), as well as *Candida krusei* and *C. albicans*, suggesting that they might be potential alternatives to antibiotics [46]. The in-silico and in-vitro studies demonstrated the immense antibacterial potential of *F. oxysporum*’s AgNPs against *P. aeruginosa* and *E. coli* [45]. The AgNPs synthesized using nitrate reductase purified from *F. oxysporum* IRAN-31C showed potent antimicrobial potential towards a wide array of human pathogenic bacteria and fungi in the disk diffusion method [117]. A study by Ballottin et al., revealed that the cotton fibers impregnated with biogenic AgNPs synthesized from *F. oxysporum* filtrate solution possessed potent antimicrobial potential even after repeated mechanical washing cycles. This might highlight the potential use of biogenic AgNPs as an antiseptic in textiles for medical applications [118].

Moreover, a study by Hamedi et al., revealed that the existence of ammonium lowered the productivity of AgNPs using *F. oxysporum* cell-free filtrate and prohibited the nitrate reductase enzyme secretion [119]. Longhi et al., reported that the combination of AgNPs synthesized using *F. oxysporum* with FLC (fluconazole) reduced the MIC of FLC around 16 to 64 times towards planktonic cells of *C. albicans* and induced a significant dose-dependent inhibition of both initial and mature biofilms of FLC-resistant *C. albicans*. Therefore, these AgNPs could represent a new strategy for treating FLC-resistant *C. albicans* infections [49]. Additionally, the combination of simvastatin with these AgNPs demonstrated antibacterial activity towards *E. coli*-producing ESBL (extended-spectrum *β*-lactamase) and MRSA (methicillin-resistant *S. aureus*). This could be a great future alternative in bacterial infection control, where smaller doses of these AgNPs are required with the same antibacterial activity [120]. Besides, its combination with polymyxin B showed a 16-fold reduction of the MIC of polymyxin B and decreased carbapenem-resistant *Acinetobacter baumannii* viability with additive and synergic effects, as well as significantly reduced cytotoxicity towards mammalian Vero cells, indicating its pharmacological safety [121]. The AgNPs synthesized with *F. oxysporum* f.sp. *pisi* were found to have moderate adulticidal potential on *Culex quinquefasciatus* (vector of filariasis) (LC_50_ 0.4, LC_99_ 4.8, and LC_90_ 4 μL/cm^2^) after 24 h exposure [122]. The synthesized AgNPs using *F. oxysporum* aqueous extract had anticancer potential towards MCF7 (IC_50_ 14 µg/mL) that was characterized using CLSM (confocal laser scanning microscopic) technique [123]. Bawskar et al. stated that the biosynthesized AgNPs using *F. oxysporum* possessed more potent antibacterial potential towards *E. coli* and *S. aureus* than chemo-synthesized AgNPs that may be due to the protein capping and their mode of entry into the bacterial cell, which encouraged biosynthetic method over the chemosynthetic one in AgNPs synthesis [124]. Two types of AgNPs, phyto-synthesized and myco-synthesized NPs were biosynthesized by AgNO_3_ reduction with *Azadirachta indica* extract and *F. oxysporum* cell filtrate, respectively that possessed lower cytotoxic potential on C26 and HaCaT cell lines as compared with citrate coated AgNPs [125]. Santos et al. proved that *F. oxysporum*-biosynthesized AgNPs without pluronic F68 (stabilizing agent) had high antibacterial potential towards *E. coli*, *P. aeruginosa*, and *S. aureus*. On the contrary, chemo-synthesized AgNPs exhibited synergism in antibacterial activity in the presence of pluronic F68 [126]. *Streptococcus agalactiae* is an important cause of invasive diseases, mainly in newborns, pregnant women, and elderly individuals [127]. The combination of *F. oxysporum*-produced AgNPs (AgNPbio) and eugenol led to a remarkable synergistic effect and significant reduction of the MIC values of both eugenol and AgNPbio towards planktonic cells of *S. agalactiae* [127]. Thakker et al., reported the synthesis of GNPs (gold nanoparticles) using *F. oxysporum* f. sp. *cubense* JT1 that showed antibacterial potential versus *Pseudomonas* sp. [128]. Moreover, the conjugated GNPs with tetracycline demonstrated powerful antibacterial activity against Gram-negative and -positive bacteria in comparison to tetracycline and free GNPs. Therefore, tetracycline conjugation with these GNPs enhanced the antibacterial potential, which may have significant therapeutic applications [129]. Yahyaei and Pourali studied the conjugation of GNPs with chemotherapeutic agents such as paclitaxel, tamoxifen, and capecitabine. Moreover, the cytotoxic effect of conjugated GNPs was assessed towards MCF7 and AGS cell lines, using MTT assay. Unlike the paclitaxel conjugated GNPs, the tamoxifen and capecitabine conjugated GNPs revealed no toxic effects due to their low half-lives and deactivation [130]. Further, Syed and Ahmad reported the synthesis of stable extracellular platinum nanoparticles, using *F. oxysporum* [131]. CdSe (cadmium/selenium) quantum dots are often used in industry as fluorescent materials. Kumar et al., and Yamaguchi et al., reported the synthesis of highly luminescent CdSe quantum dots by *F. oxysporum* [132,133]. In 2013, Syed and Ahmad synthesized highly fluorescent CdTe quantum dots using *F. oxysporum* at ambient conditions by the reaction with a mixture of TeCl_4_ and CdCl_2_. These nanoparticles exhibited antibacterial potential towards Gram-negative and -positive bacteria [53]. Riddin et al., analyzed the biosynthesized platinum (Pt) nanoparticles by *F. oxysporum* f. sp. *lycopersici* at both intercellular and extracellular levels. It was found that only the extracellular nanoparticle production was proved to be statistically significant with a yield of 4.85 mg/L [134].

### 2.2. Metal Sulfide Nanoparticles

In addition, Q-state CdS NPs were biosynthesized by the reaction of aqueous CdSO_4_ solution with *F. oxysporum* [135]. The chemically-synthesized CdSQDs inhibited *E. coli* cell proliferation in a dose-dependent manner, unlike the biogenic CdSQDs synthesized by *F. oxysporum* f. sp. *lycopersici*, which showed an antibacterial potential only at high concentration. Additionally, only the biogenic CdSQDs showed no inhibition on seed germination after incubation of biogenic and chemical CdSQDs with *Lactuca sativa* seeds [43]. Bi_2_S_3_ (bismuth sulfide) NPs have significantly varied applications, including photodiode arrays, photovoltaic materials, and bio-imaging. Uddin et al., synthesized a highly fluorescent, natural protein capped Bi_2_S_3_NPs by subjecting *F. oxysporum* to bismuth nitrate penta-hydrate, along with sodium sulfite under ambient conditions of pressure, temperature, and pH. It was found that they were fundamentally much more fluorescent than fluorophores (toxic fluorescent chemical compounds), which are largely utilized in immunohistochemistry, imaging, and biochemistry [48].

### 2.3. Metal Oxide Nanoparticles

It was reported that *F. oxysporum* might have vast commercial implications in low-cost, room-temperature, ecofriendly syntheses of technologically significant oxide nanomaterials from available potentially cheap naturally raw materials [136]. *F. oxysporum* rapidly bio-transformed the naturally occurring amorphous biosilica in rice husk into crystalline silica NPs. This could lead to an economically viable and energy-conserving green approach toward the large-scale synthesis of oxide nanomaterials [136]. Moreover, the mesophilic *F. oxysporum* bioleached Fly-ash at ambient conditions produced highly stable, crystalline, fluorescent, water-soluble, and protein-capped silica nanoparticles [52]. It was found that *F. oxysporum* enriched zirconia in zircon sand by a process of selective extracellular bioleaching of silica nanoparticles. It was proposed that the fungal enzymes specifically hydrolyzed the silicates in the sand to form silicic acid, which on condensation by certain other fungal enzymes resulted in silica nanoparticles synthesis at room temperature [136]. A water dispersible and thermo-stable Ag/Ag_2_O NPs were produced from silver oxide micro-powder using *F. oxysporum*. These Ag/Ag_2_O NPs may become a potential candidate for enzyme-free glucose determination and exhibited catalytic potency for MB (methylene blue) degradation in presence of NaBH_4_ (reducing agent). Additionally, they showed an excellent antimicrobial potential against *A. niger* and *B. subtilis* [137].

## 3. Biotechnological and Industrial Relevance of *F. oxysporum*

*F. oxysporum* is a wealthy source of enzymes with significant biotechnological and industrial potential. In various studies, *F. oxysporum* demonstrated a remarkably high enzymatic performance and the ability to degrade different biomasses. Herein, the reported enzymes from *F. oxysporum* and their industrial and biotechnological applications are highlighted.

### 3.1. F. oxysporum Enzymes and Their Applications

#### 3.1.1. Glycoside Hydrolases

Cellulases are accountable for cellulose hydrolysis, including *β*-1,4-endoglucanase, cellobiohydrolase, and *β*-glucosidases (BGL), which catalyze the hydrolysis of aryl- and alkyl-*β*-glucosides, as well as oligosaccharides and diglucosides [1,3,5]. Cellulases’ preparations have been added to the ruminant animals’ diets to stimulate feed processing and fiber digestion to increase the extent and rate of digestion [138]. Zhao et al., purified extracellular BGL from *F. oxysporum* that had high acid stability (pH 3) and cellobiose hydrolytic activity relative to Celluclast^®^ (commercial cellulase). Its supplementation also released more reducing sugars (330 mg/g substrate) from cellulose, in comparison to Novozymes (commercial BGL, 267 mg/g substrate) under simulated gastric conditions. Thus, it could be a good source for a new commercial BGL for improving the feed and food quality in the animal feed industry and could be used in combination with Celluclast for industrial applications that required degradation of cellulose at acidic pH [37].

Fucose is a low abundant deoxy-hexose sugar, usually attached to the non-reducing ends of oligolipids, oligosaccharides, and other glycoconjugates (e.g., immunoglobulins, glycoproteins, blood group substances, and mucins). Besides, it is a component of marine algal polysaccharides, human milk oligosaccharides, and plant gums [139]. FUC (*α*-L-fucosidase) a glycoside hydrolase, catalyzes the breakdown of the terminal *α*-L-fucosidic bonds. It has remarkable roles in various bioprocesses, and it is used as a marker for hepatocellular carcinoma detection and structural analyses of complex natural products [140]. *F. oxysporum* produced FUC in large amounts through induction by L-fucose. This enzyme hydrolyzed *p*-nitrophenyl *α*-L-fucoside (synthetic substrate) like marine gastropod and mammalian enzymes, thus it could replace these enzymes. It had beneficial use as an analytical tool for the structural elucidation of complex carbohydrates and oligosaccharides [41]. Additionally, Yano et al., purified a novel FUC from *F. oxysporum* culture broth. Besides nitrophenyl compounds, this enzyme had a novel substrate specificity. It could hydrolyze porcine mucin and blood group substances [42].

α-D-Galactopyranosidase (GPase) is a glycoside hydrolase that hydrolyzes the α-galactopyranosyl linkages at non-reducing ends of sugar chains. They are utilized for various applications, including eliminating non-digestible oligosaccharides such as stachyose in legume products and soybean, improving the digestibility of animal feed, and increasing the yield and quality of sucrose in sugar refineries [141]. FoAP1 and FoAP2 are two bifunctional enzymes that were isolated and characterized from the culture supernatant of *F. oxysporum* 12S, possessing GPase (α-D-galactopyranosidase)/APase (β-L-arabinopyranosidase) activities in a ratio 1.7 and 0.2, respectively using PNP-α-D-Galp (para-nitrophenyl α-D-galactopyranoside) and PNP-β-L-Arap (para-nitrophenyl α-l-arabinopyranoside) as substrates [38]. A novel GPase, FoGP1 was purified from *F. oxysporum*, exhibiting degrading activity with terminal α-1,3-galactosyl linkages in gum Arabic side chains. Therefore, it might be used for improving gum Arabic physical properties, which is an industrially important polysaccharide used as a coating agent and an emulsion stabilizer [34].

Xylan is one of the most abundant carbohydrates on earth. Its complete degradation is accomplished by the action of various enzymes such as β-D-xylosidases and endo-β-1,4-xylanases. Alconada and Martínez characterized extracellular β-xylosidase and endo-1,4-β-xylanase from *F. oxysporum* f. sp. *melonis*, growing in a medium containing oat spelt xylan. The latter had a high affinity towards oat spelt xylan [142]. Additionally, FoXyn10a, new GH10 xylanase was purified and structurally characterized from *F. oxysporum* [143]. Anasontzis et al., reported that the constitutive homologous overexpression of the endo-xylanase in *F. oxysporum* increased ethanol production during CBP of lignocellulosics [144]. *Xyn11a* is an endo-1,4-β-xylanase gene from *F. oxysporum*, belonging to the fungal glycosyl hydrolase family 11 (GH-11) that was cloned and expressed in *Pichia pastoris*. Recombinant *P. pastoris* possessed efficient xylanase secreting potential and a high level of enzymatic activity under methanol induction [145]. Gómez et al., identified Xyl2, an endo-β-1,4-xylanase from *F. oxysporum*. It was highly active at alkaline pH and its immobilization on certain supports significantly increased its thermal stability. Together, these properties rendered Xyl2, an attractive biocatalyst for the sustainable industrial degradation of xylan [146]. Najjarzadeh et al., compared xylanase production by different inducers, such as lactose, sophorose, xylooligosaccharides, and cellooligosaccharides in *F. oxysporum* f. sp. *lycopersici*. It was found that xylooligo-saccharides were more effective than other substrates at the induction of β-xylosidases and endoxylanases. Moreover, xylotetraose, xylohexaose, and xylobiose were the best inducers of endoxylanase, extracellular β-xylosidase, and cell-bound β-xylosidase, respectively [147].

#### 3.1.2. Nitrilases

Microbial nitrilases are biocatalysts of remarkable organic importance in terms of nitrile conversion. Nitrile compounds include aromatic and simple aliphatic metabolites, cyano-lipids, and cyano-glucosides which serve as key intermediates and compounds in various biochemical pathways [148]. Processes involving enzymatic conversion of nitrile substrates to higher value amides and carboxylic acid groups are preferred over the chemical synthesis for their production of fewer harmful reaction by-products and greater reaction specificity [149]. Industrial application of nitrile-converting enzymes includes acrylamide and nicotinamide production [35]. The newly isolated nitrilase from *F. oxysporum* f. sp. *lycopercisi* ED-3 strain had a wide substrate specificity toward *ortho*-substituted heterocyclic, aliphatic, and aromatic nitriles and had optimal activity at temperature 50 °C and pH 7.0 [35].

#### 3.1.3. Nitric Oxide Reductases

Denitrification is a substantial process in the nitrogen cycle, which involves the reduction of NO^−2^ and/or NO^3−^ to either N_2_O or N_2_. This process can be performed by many bacteria, as well as fungi through a series of consecutive metallo- enzyme-catalyzed chemical reactions [150]. It is a reversible process of nitrogen fixation, where it carries back the fixed N_2_ to the atmosphere. It was found that the main source of global N_2_O emissions is the microbial activities of denitrification and nitrification [151]. Therefore, controlling microbial denitrification is most important for N_2_O emission reduction [152]. It was reported that *F. oxysporum* exhibited a distinct denitrifying potential that resulted in the anaerobic evolution of N_2_O from NO^−2^ and NO^3−^ [153]. Further, a nitric oxide reductase (NOR), cytochrome P450nor, belonging to P450 superfamily was purified from *F. oxysporum*, which exhibited remarkable NO reduction potential [36,150,152]. It showed a unique bio-function compared with other usual P450s. While the usual P450s are involved in metabolizing various biological substances through mono-oxygenation reaction using O_2_, P450nor catalyzed the NO reduction but not the mono-oxygenation [150,154]. Shoun and Tanimoto described a heme-thiolate protein or P450 from *F. oxysporum* that was involved in the NO (nitrogen monoxide) reduction to N_2_O (dinitrogen oxide) [153]. In contrast to other bacterial cytochrome *bc*-containing NO reductases, it did not need a flavoprotein for electron transfer from NADH to the heme, but it utilized NADH directly for the reduction process [155]. Additionally, Daiber et al., identified P450nor (cytochrome P450 NADH-NO), a heme-thiolate protein that catalyzed the reduction of two NO molecules to N_2_O. P450nor was observed to have a remarkable role in protecting the fungus from NO inhibition of mitochondria [156].

#### 3.1.4. Cutinases

Esters having a chain of fewer than 10 carbon atoms are used as flavor compounds in the pharmaceutical, cosmetic, and food industries [157]. Natural synthesis of flavor compounds takes place either by enzymatic bioconversion or by microorganisms, the former path was found to be an easier and more suitable method [157]. The high demand of various industries for fatty acid ester leads to possible growth of the market to $2.44 billion by 2022, from $1.83 billion in 2014 [33,158]. Enzyme immobilization increases their stability and allows their easy separation from the reaction and reuse to overcome the drawbacks of utilizing enzymes such as low operational or storage stability, and/or heat and organic solvent sensitivity [159]. The im*Focut*5a a CLEAs (cross-linked enzyme aggregates) was produced from a crude *F. oxysporum* cutinase preparation. This immobilized cutinase possessed a remarkable thermo-stability and was able to synthesize butyl butyrate (pineapple flavor) at a high yield of bioconversion (99%) through the *trans*-esterification of vinyl butyrate with butanol. This bioconversion presented an eco-friendly and sustainable production of natural flavor compounds, underpinning its industrial potential for use in food bioprocesses [33]. FoCut5a, a cutinase was purified from *F. oxysporum* and expressed either in the periplasm or cytoplasm of *E. coli* BL21. It could hydrolyze PET (polyethylene terephthalate) and synthetic polymers. Therefore, it could be used in industrial applications as a biocatalyst for the eco-friendly treatment of synthetic polymers [160].

#### 3.1.5. Fructosyl Amino Acid Oxidases

Glycation is the non-enzymatic glycosylation of proteins due to the condensation of reducing sugars such as glucose with the proteins (α- or ε-amino groups) to form a Schiff’s base [161]. Glycation leads to browning of foods during long-term storage, which represents a problem in the food industry [39]. Glycation of hemoglobin, blood proteins, and albumin was found to be enhanced in diabetic patients. Glycated proteins, particularly glycated hemoglobin A1c, are important markers for assessing the effectiveness of anti-diabetic agents. Fructosyl amino acid oxidase (FAOD) based assays have become an attractive alternative to conventional detection methods for measuring glycated proteins [161]. Sakai et al., purified FLO (fructosyl lysine oxidase) from *F. oxysporum* S-lF4 that acted against fructosyl poly L-lysine. FLOD could be used for measuring glycated proteins such as glycated albumin in the serum [39].

#### 3.1.6. Lipoxygenase

Lipoxygenase (LOX) is a dioxygenase that catalyzes the hydro-peroxidation of polyunsaturated fatty acids such as arachidonic and linoleic acids [162]. It is expressed in epithelial, tumor, and immune cells that have various physiological functions such as skin disorders, inflammation, and tumorigenesis [163]. Bisakowski et al., extracted and purified LOX from *F. oxysporum* that shared many of the characteristics with LOXs reported from other sources such as substrate specificity, pH, enzyme inhibition, activation, and other kinetic studies [40].

#### 3.1.7. Laccases

Laccases are belonging to oxidoreductases that catalyze the O_2_ reduction to H_2_O with simultaneous organic substrates oxidation. They oxidize phenolic substrates but are also able to oxidize bigger or non-phenolic substrates by LMS (laccase-mediator system), where a small phenolic compound acts as a mediator [164]. Additionally, they have been reported as potential lignin-degrading enzymes and as solubilizing agents [165,166]. They have attracted great interest because of their wide applications in diverse biotechnological and industrial fields such as textile dye decolorization, pulp bleaching, organic synthesis, bioremediation, and detoxification of environmental pollutants, delignification, or biofuel production [1,3,5].

Kwiatos et al., expressed *F. oxysporum* Gr2 laccase in *Saccharomyces cerevisiae* and engineered it for getting higher effect against 2,6-dimethoxyphenol and higher expression levels. The resulted laccase had a promising potential for different industrial uses such as solubilization of brown coal, which is a clean coal technology, aiming at converting lignite to its cleaner form [164]. In 2018, Kwiatos et al., reported that *F. oxysporum* LOCK-1134 isolated from brown coal, efficiently bio-solubilized lignite, producing liquefied products that had over 99% less Hg and 50% less sulfur than the crude coal. Additionally, its laccase was expressed in *Pichia pastoris*. The resulted novel laccase improved the biodegradation process in presence of LMS. It released fulvic and humic acids from liquefied coal. The latter are environmentally friendly fertilizers that possessed a stimulating influence on crop growth [165].

#### 3.1.8. Aromatic Carboxylic Acid Decarboxylases

The non-oxidative aromatic carboxylic acid decarboxylases catalyze the reversible decarboxylation of phenolic carboxylic acids. Therefore, they are useful biocatalysts for preparing high-value phenolic compounds by the decarboxylation of phenolic carboxylic acids derived from lignin, which opens up a new prospect for high-value utilization of the world second most abundant organic substance [167,168]. Song et al., characterized 2,3-DHBD_Fo, a 2,3-dihydroxybenzoic acid decarboxylase from *F. oxysporum* that possessed a relatively high catalytic decarboxylation efficiency for DHBA (2,3-dihydroxybenzoic acid) and catechol, hence it had a different substrate spectrum from other benzoic acid decarboxylases [167].

#### 3.1.9. Keratinases

Keratins are complex proteins that formed of β-sheets and α-helix structures. They are commonly found in agro-industrial residues such as swine hair and chicken feathers. Keratinous wastes are treated in non-eco-friendly ways, including landfills and incinerators [169,170]. *F. oxysporum* isolated from chicken feathers showed potential for keratinase production that had the highest degradation percentage (59.20% *w/w*) in swine hair [169].

#### 3.1.10. Phospholipase B

Phospholipase B (PLB) hydrolyzes the phospholipid acyl groups to produce fatty acids and phosphoglycerates [171]. PLB is utilized to produce beneficial phospholipid derivatives, reduce food’s cholesterol content, and refine vegetable oils, especially in terms of crude oil degumming [172]. Su et al., characterized a putative lipase from *F. oxysporum* NCBI-EGU84973.1 that was expressed in *P. pastoris* and classified as a PLB. It had phospholipids hydrolyzing potential greater than its lipase capacity where it hydrolyzed the fatty acyl ester bond at the *sn*-1 and -2 positions of the phospholipids and reduced the oil phosphorus contents. This proved the potential industrial use of this PLB in oil degumming applications [172].

#### 3.1.11. Triosephosphate Isomerase

TPI (triosephosphate isomerase) is a glycolysis enzyme that catalyzes the reversible isomerization between DHAP (dihydroxyactetone-3-phosphate) and GAP (glyceraldehyde-3-phosphate) [173]. Therefore, TPI is essential for pathogenic organisms to get the energy needed for survival and infection. Hernández-Ochoa et al., isolated, cloned, and overexpressed *Tpi* gene from *F. oxysporum* isolated from a wild species collected from a bean crop. They purified FoxTPI recombinant protein that had the TPIs classical topology conserved in other organisms [14].

### 3.2. Applications of F. oxysporum

Biodiesel (biofuel) is obtained from renewable sources such as animal fat or vegetable oil by *trans*-esterification of triglycerides to give fatty acid alkyl esters [174]. It is used as a full or partial substitute for petrol diesel in combustion engines [175]. Its production attracts attention worldwide due to the environmental benefits such as biodegradation that reduced the emission of sulfur and aromatic hydrocarbons during fuel combustion and decreased emission of CO_2_, CO, and particulate materials. The accumulated lipids in microorganisms such as algae, fungi, and bacteria are mainly triacylglycerols (TAG) that are utilized as metabolites for biodiesel production [32]. *F. oxysporum* NRC2017 isolated from Egyptian soil had remarkable lipid producing capacity (55.2%). It showed the highest lipid accumulation 98.3 mg/g in the presence of baggase and its fatty acids were found to be suitable for biodiesel production based on GC analysis [32].

On earth, the most abundant source of biomass is lignocellulosic material that includes agricultural residues, grasses, wood, or any non-food-plant sources. Its microbial fermentation produces ethanol and other solvents that represent an alternative path for wastes treatment and production of fuel additives and chemical feedstocks. *F. oxysporum* was found to have the potential for converting D-xylose, as well as cellulose to ethanol in a one-step process, indicating its capacity for ethanol production [176].

Bioethanol production is a harsh operational process that needs potent biocatalysts. CBP (consolidated bioprocessing) is an economical and efficient method of manufacturing bioethanol from lignocellulose. CBP integrates the fermentation and hydrolysis steps into a single process, leading to a significant reduction in the steps of the biorefining process. Ali et al., reported that *F. oxysporum* had a high potential for CBP of lignocellulose to bioethanol and it could be a commercially competitive CBP agent [177]. It was observed a significant inter-strain divergence regarding the capacity of different *F. oxysporum* strains to produce alcohol from wheat straw [178]. Nait M’Barek et al., assessed the potential of *F. oxysporum* for bioethanol production from non-valorized OMW (olive mill waste) using CBP. It showed maximum bioethanol yield and production of 0.84 g/g and 2.47 g/L, respectively, indicating its importance as a bio-agent for single-pot local bio-refinery [179]. Moreover, *F. oxysporum* BN converted imidazolium-based ionic liquid (IL)-pretreated rice straw to bioethanol via CBP with 64.2% of the theoretical yield of 0.125 g ethanol/g rice straw [164]. It secreted a novel IL-tolerant cellulase that can direct the conversion of IL-pretreated lignocellulose residue to ethanol, which had a significant potential to bring a breakthrough in commercial ethanol production by the reduction of the overall cost [180].

## 4. Secondary Metabolites from *F. oxysporum* and Their Bioactivities

### 4.1. Anthranilates

Anthranilates are derivatives of anthranilic acid that constitute an important part of several bio-metabolites and serve as a scaffold for developing remarkable pharmaceuticals for the management of the pathogenesis and pathophysiology of diverse disorders. They possessed impressive bioactivates such as antiviral, antimicrobial, insecticidal, anti-inflammatory, anti-diabetic, and anticancer [181]. Compounds **1**–**21** are anthranilic acid derivatives that had been purified and characterized only from *F. oxysporum* f. sp. *dianthi* extracts using HPLC-, pyrolysis-, and HR-MS [56]. It was reported that the anthranilic acid derivatives are originated from **2** that is formed from benzoate and anthranilate [182]. Subsequently, it undergoes hydroxylation at C-2′ to produce dianthalexin, hydroxylation at C-4 to yield **3** or **5**, and methylation to **9** or **8**. Moreover, **20** and **19** are produced from **5** and **3** [56,183] (Scheme 1, Figure 1).

### 4.2. Fumonisins

Fumonisins are mycotoxins, belonging to fungal polyketides. They have two propane-1,2,3-tricarboxylic acid chains esterified to an aminopolyol skeleton [184]. They inhibit a ceramide synthase, the key enzyme in the biosynthetic pathway of sphingolipids, leading to serious mycotoxicoses [57,185]. Fumonisin derivatives **22**–**30** were isolated from *F. oxysporum* associated with *Asparagus officinalis* and *Dianthus caryophyllus*. They were characterized by FABMS, ES-LCMS, and NMR techniques [57,58,59,60] (Figure 2).

### 4.3. Jasmonates

Jasmonates are lipid-based metabolites, possessing jasmonic acid (3-oxo-2-(pent-2′-enyl)cyclopentane acetic acid) framework that are found in fungi, bacteria, and plants [186,187]. In plants, they function as growth regulators and play major roles in the defense of plants against insects and diseases [187]. In addition, hydroxylated jasmonic acids, unsaturated or saturated, and *cis*- or *trans*-configured elongated side-chain derivatives were reported from fungi. They can also form conjugates with amino acids such as isoleucine. In a study by Miersch et al., jasmonates derivatives **31**–**52** were purified from *F. oxysporum* f. sp. *matthiolae* using RP-18 Lichrolut, DEAE-Sephadex-A25, and 100-C18 Eurospher and characterized by GC-MS and HPLC [61] (Figure 3 and Figure 4).

### 4.4. Alkaloids

The reported studies revealed the isolation of diverse classes of alkaloids from *F. oxysporum.* Oxysporidinone (**53**), a novel 3,5-disubstituted *N*-methyl-4-hydroxy-2-pyridone was purified from *F. oxysporum* (CBS 330.95) culture by counter-current and SiO_2_ CC and characterized by UV, NMR, IR, and MS tools. It had growth inhibitory potential towards phyto-pathogenic fungi; *Aspergillus niger*, *Botrytis cinerea*, *Alternaria alternata*, and *Venturia inequalis* (MICs 10, 1, 50, and 10 µg/mL, respectively) (Table 2). It showed no observable activity towards *B. subtilis*, *P. aeruginosa*, *C. albicans*, and *S. cervisiae* (conc. 1 mg/mL) [62]. Bioassay-guided separation of *F. oxysporum* (N17B) extract gave three new *N*-methyl-4-hydroxy-2-pyridinone derivatives; 6-epi-oxysporidinone (**59**), dimethyl ketal of oxysporidinone (**60**), and N-demethylsambutoxin (**61**), along with (−)-oxysporidinone (**54**) and (−)-sambutoxin (**58**) that were identified by NMR and MS techniques [63] (Figure 5). Compound **54** was identical to **53** but with the opposite sign of optical rotation. Compound **59** was a 6′-hydroxy epimer of **53**, however, **61** was similar to **58** with the lack of N-CH_3_ group. Compound **58** was isolated previously as a hemorrhagic mycotoxin from *F. sambucinum* [188]. Interestingly, **54** (IC_50_ 2.0 µg/mL) had a powerful fungistatic potential towards *A. fumigatus*, whereas its epimer **59** (IC_50_ 35.0 µg/mL) showed a marginal effect, compared with amphotericin B (IC_50_ 0.91 µg/mL) in the MABA (microplate Alamar blue assay). Whilst other compounds were inactive [63]. (−)-4,6′-Anhydrooxysporidinone (**57**) isolated from EtOAc extract of a solid endophytic fungus *F. oxysporum*, had moderate anti-BS (*Bacillus subtilis*) activity (MIC 25.0 μg/mL) and weak anti-MRSA potential (MIC 100 μg/mL) [65]. On the other side, (−)-oxysporidinone (**54**), (−)-6-deoxyoxysporidinone (**56**), and (−)-4,6′-anhydrooxysporidinone (**57**) isolated from *F. oxysporum* EPH2R_AA,_ harboring *Ephedra fasciculata* had no cytotoxic activities towards NCI-H460, MIA Pa Ca-2, MCF-7, and SF-268 [64]. Fusaric acid (**63**) was identified as antioomycete metabolite from *F. oxysporum* EF119 by MS and NMR analyses. It possessed in vivo and in vitro antioomycete potential against *P*. *capsici* (causative agent of wheat leaf rust) and *P. infestans* (causative agent of tomato late blight) with IC_50_ values < 1 µg/mL. It also completely suppressed the growth of various bacteria (IC_50_ values ranged from 0.2 to 12 µg/mL, Conc. < 100 µg/mL). Thus, it could be used as a biocontrol agent towards tomato late blight produced by *P. infestans* [66]. Moreover, **63** was identified from *F. oxysporum* isolated from an infected grapevine. It induced extensive necrosis formation on tobacco in the leaf-puncture assay at 0.5 mg/mL. Fusaric acid is a nonspecific toxin produced by many *Fusarium* species, and usually is the main phytotoxin [67]. The new fusaric acid derivatives, fusaricates A-G (**65**–**71**) and 10-hydroxy-11-chlorofusaric acid (**72**) purified from *F. oxysporum* isolated from *Drepanocarpus lunatus* fruits had fusaric acid linked to a polyalcohol moiety through an ester bond (Figure 6). Their structures were elucidated by NMR and MS data and the absolute configuration was established using chiral GC-MS. Only **72** had weak cytotoxicity against L5178Y (IC_50_ 37.7 µM), compared to kahalalide F (IC_50_ 4.3 µM).

Compounds **65**, **68**, **69** and **72** showed phytotoxicity towards barley leaves using cotton cotyledonary leaf bioassay almost equal to that of fusaric acid, suggesting their promising potential in organic farming. It was suggested that the existence of the C-10-hydroxyl group or C-10 and C-11 double bond led to a decrease in phytotoxicity [69]. Liu et al., postulated the biosynthetic pathway of fusaricates A-G (**65**–**71**). Isotopic ^13^C and ^14^C tracer studies revealed that C-2 to C-4 and C-7 of fusaric acid were originated from oxaloacetate, while C-5, C-6, and C-8 to C-11 were derived from three acetate units [189,190]. The nitrogen atom of pyridine ring is originated mainly from glutamine [191]. The formation of ester linkage between polyalcohols and fusaric acid could be catalyzed by lipases [192] (Scheme 2). 

Nenkep et al., isolated a polycyclic quinazoline alkaloid, oxysporizoline (**73**), in addition to 1*H*-indol-3-butanamide (**74**) and butenolide (**75**) from marine-mudflat-derived *F. oxysporum*. Compounds **73** and **75** displayed weak antibacterial activity against MRSA (MIC 6.25 µg/mL). They also exhibited potent DPPH radical scavenging potential (IC_50_ 10 and 12 μM, respectively) than ascorbic acid (IC_50_, 20 μM) [70] (Figure 6).

2-Oxo-8-azatricyclo [9.3.1.1^3,7^]-hexadeca-1(15),3(16),4,6,11,13-hexaen-10-one (**80**) isolated from *F. oxysporum*, exhibited weak activity towards MCF-7, PC-3, and A549 and potent antimicrobial effect more than controls against various tested microorganisms with MIC values ranging from 0.8–12.5 µg/mL [73] (Figure 7). Fusarioxazin (**81**) was separated from *F. oxysporum* associated with *Vicia faba* roots. It displayed a significant cytotoxic effect toward HCT-116, MCF-7, and A549 cell lines (IC_50_s 2.1, 1.8, and 3.2 µM, respectively), in comparison to doxorubicin (IC_50_s 0.68, 0.54, and 0.39 µM, respectively). Additionally, it possessed antibacterial potential towards *S. aureus* (IZD 14.8 mm and MIC 5.3 mg/mL) and *B. cereus* (MIC 3.7 mg/mL and IZD 18.9 mm), in comparison to ciprofloxacin (IZDs 16.9 and 20.5 mm; MICs 3.9 and 2.3 mg/mL, respectively) [18]. Epi-trichosetin A (**82**), a new tetramic acid derivative, along with trichosetin (**83**) were separated by SiO_2_ and Rp-HPLC from *F. oxysporum* FKI-4553 broth. Compound **82** was a C-5′ epimer of **83**. Compounds **82** (IC_50_ 83 µM) and **83** (IC_50_ 30 µM) inhibited UPP (undecaprenyl pyrophosphate) synthase activity of *S. aureus*. They had a broad antibacterial effect, in particular potent effect against Gram-positive bacteria, including MSSA (methicillin-sensitive *S. aureus*) and MRSA, where **82** appeared to be more potent than **83** in the paper disk method [74]. Kumar et al. purified vinblastine (**86**) and vincristine (**87**) from *F. oxysporum* isolated from *Catharanthus roseus* using preparative TLC and HPLC (Figure 8). They were characterized by UV-Vis, ESIMS, and NMR spectroscopy [76].

### 4.5. Cyclic Peptides and Depsipeptides

*F. oxysporum* yielded bioactive cyclic depsipeptides such as enniatins (ENs) and beauvericin (BEA, **99**) (Figure 9 and Figure 10). ENs are characterized by an alternating sequence of three D-α-hydroxyisovaleric acids and three N-methyl-L-amino acids in their structure. ENs H (**96**), I (**97**), and MK1688 (**98**), and BEA (**99**) were purified from *F. oxysporum* KFCC-11363P submerged cultures chloroform extracts using HPLC and assessed for cytotoxicity towards A549, SK-OV-3, SK-MEL-2, and HCT15 in the SRB (sulforhodamine B). Compound **97** (EC_50_ 0.49–0.53 µM) and MK1688 (EC_50_ 0.45–0.63 µM) exhibited the most potent growth inhibition towards the tested cell lines that were three- to four-fold more than that of **99** and **96**. On the other side, **96** and **99** exhibited powerful activity towards SK-OV-3, A549, SK-MEL-2, and HCT15 cells (EC_50_ 1.71–2.45 µM for **96** and 1.39–1.86 µM for **99**) [83]. ENs cytotoxic potential could be attributed to their ionophorous behavior, since altering the ions transport across membranes may lead to disruption of the cationic selectivity of the cell wall and induction of cell death by apoptosis accompanied by DNA fragmentation [83]. ENs A (**91**), A1 (**92**), and B1 (**93**) isolated from *F. oxysporum* N17B exhibited moderate effectiveness towards *C. albicans*, *C. neoformans*, and *M. intracellulare* with IC_50_ values ranging from 2.0 to 50.0 µg/mL in the MABA [63]. Moreover, Wang et al., reported that **99** isolated from *F. oxysporum* obtained from *C. kanehirae* bark exhibited cytotoxic potential towards A549, PC-3, and PANC-1 (IC_50_s 10.4, 49.5, and 47.2 μM, respectively), in comparison to cisplatin (IC_50_s 19.8, 26.8, and 26.2 μM, respectively) in the MTT method. It also had strong anti-MRSA and anti-BS potential (MIC 3.125 μg/mL) in the microtiter plate assay [65].

Further, **99** was obtained from the EtOAc extracts of *F. oxysporum* SS46 and SS50 associated with *Smallanthus sonchifolius*. It showed cytotoxicity against MDA-MB435, HCT-8, and SF295 (IC_50_s 3.17, 3.02, and 2.39 μg/mL, respectively), compared to doxorubicin (IC_50_ 0.2, 0.04, and 0.04 μg/mL, respectively) in the MTT assay. Additionally, it had potent in vitro leishmanicidal potential (EC_50_ 1.86 μM) towards *Leishmania braziliensis*, compared to geneticin (EC_50_ 0.007 μM) [84]. Zhan et al., revealed that **99** was cytotoxic towards NCI-H460, MIA Pa Ca-2, MCF-7, and SF-268 (IC_50_s 1.41–2.29 µM, respectively), compared with doxorubicin (IC_50_ 0.01–0.07 µM) [64]. 

Furthermore, it prohibited migration of MDA-MB-231 and PC-3M cells (conc. ranging from 3.0 to 4.0 µM and 2.0 to 2.5, respectively) in the WHA (wound healing assay). NIH ImageJ software and WHA suggested that **99** was able to inhibit PC-3M and MDA-MB-231 migration at sub-lethal concentrations. Moreover, it possessed potent antiangiogenic potential at sub-lethal concentrations, as indicated by complete inhibition of HUVEC-2 network formation at 3.0 µM below IC_25_ (5.0 µM) and IC_50_ (7.5 µM) [64]. Cyclosporine A (**100**) was isolated from mycelia extract of non-pathogenic *F. oxysporum* S6 using reversed-phase silica gel and HPLC. It prohibited the growth and suppressed sclerotia formation of the phytopathogenic fungus *Sclerotinia sclerotiorum* with MIC 0.1 µg/disc that made it suitable to be utilized as a bio-fungicide. Moreover, a remarkable increase in the number of surviving soybean plants was noted when *F. oxysporum* and *S. sclerotiorum* were inoculated together, in comparison to plants inoculated with *S. sclerotiorum* alone in the greenhouse assay. Hence, *F. oxysporum* could be a good biocontrol agent for *S. sclerotiorum* in soybean because of its metabolite **100** that was responsible for the in vitro antagonistic activity [86]. It is noteworthy to mention that some *F. oxysporum* strains could repress the growth of *Pythium ultimum* in cucumber [193] and affected *S. sclerotiorum* sclerotia germination [194].

### 4.6. Glucosylceramides

Glucosylceramides (GCs) are neutral glycosphingolipids, having glucose in 1-*O*-*β*-glycosidic linkage with a ceramide [195]. Bernardino et al., isolated and purified the GCs, **102**–**104** from *F. oxysporum*. These GCs were assessed for their potential in inducing resistance in *Nicotiana tabacum cv* Xanthi plants against TMV (Tobacco mosaic virus) (Figure 11). Spraying tobacco plants with GCs before virus infection reduced the incidence of necrotic lesions caused by TMV. After GCs treatment, the infected plants with the virus exhibited a reduction in HR (hypersensitive response) lesions, indicating GCs antiviral effect. The results revealed that GCs stimulated the early accumulation of H_2_O_2_ and superoxide radicals, which act as a plant immunity elicitor to combat diseases influencing the plants [87].

### 4.7. Quinones

Chromatographic separation of *F*. *oxysporum* f. sp. *ciceris* ITCC-3636 EtOAc extract afforded anhydrofusarubin (**105**), fusarubin (**107**), 8-*O*-methylfusarubin (**108**), and 3-*O*-methyl-8-*O*-methylfusarubin (**109**) that were elucidated by LC/ESI-MS and detailed NMR spectra (Figure 12). The EtOAc extract had strong anti-nemic activity towards *Meloidogyne incognita* (LC_50_ 56.2 μg/mL) than n-BuOH fraction (LC_50_ 97.4 μg/mL), while they were moderately active versus *Rotylenchulus reniformis* (LC_50_ 134.5–189.2 μg/mL). All metabolites exhibited high anti-nemic potential towards both nematodes (LC_50_ ranged from 248.9 to 652.3 μg/mL). Among them, **107** showed the highest potential on both nematodes (LC_50_ 248.9 μg/mL for *M*. *incognita* and LC_50_ 301.6 μg/mL for *R*. *reniformis*), followed by **105** (LC_50_ 257.6 and 285.3 μg/mL, respectively), compared to carbofuran (LC_50_ 54.2 and 37.6 μg/mL, respectively). Whilst the methyl-substituted derivatives had moderate activity against *M. incognita* (LC_50_ ranging from 478.5 to 376.4 μg/mL) [88]. Moreover, the EtOAc extracts of *F. oxysporum* SS46 and SS50 isolated from *Smallanthus sonchifolius* yielded **105** that showed cytotoxicity against MDA-MB435, HCT-8, and SF295 (IC_50_ 6.23, 9.85, and 6.32 μg/mL, respectively), compared to doxorubicin (IC_50_ 0.2, 0.04, and 0.04 μg/mL, respectively) in the MTT assay [84]. Further, naphthoquinone derivatives, **106**–**108** and **111**–**115** were isolated from *F. oxysporum* obtained from citrus trees diseased roots. Compound **111** had strong activity towards *S. aureus* (MIC 4 µg/mL) and weak activity against *Streptococcus pyogenes* (MIC > 128 µg/mL), while **112** was moderately active against the two strains (MICs 32.0 and 64.0 µg/mL, respectively). On the other hand, **106**, **108**, **113**, and **114** showed weak activity towards *S. pyogenes* in the microdilution assay [90].

### 4.8. *Xanthone* Derivatives

Bikaverin (**119**), intensively colored pigment was reported firstly from *F. vasinfectum* and *F. lycopersici* [85,96]. It belongs to the NRPKs (non-reducing polyketides) group that is produced by type I PKS [196,197]. By genetic engineering together with HPLC-HRMS and NMR tools, Arndt et al., identified the biosynthetic way for **119** and characterized its intermediates [198] (Scheme 3). Compounds **119** and **125** were isolated from *F. oxysporum* CECIS associated with *Cylindropuntia echinocarpus* (Figure 13). They were assessed for their cytotoxic activity towards a panel of four sentinel cancer cell lines by the MTT assay. Only **119** was cytotoxic towards NCI-H460, MIA Pa Ca-2, MCF-7, and SF-268 (IC_50_ 0.26–0.43 µM), compared with doxorubicin (IC_50_ 0.01–0.07 µM). It is noteworthy that **125** that lacks the C-6-OH group did not have cytotoxic activity even at concentrations of 4.0 and 2.0 µg/mL [64]. Further, **119** isolated from *F*. *oxysporum* f. sp. *lycopersici* as a purple-colored compound, exhibited a protective effect on oxidative stress and attenuated H_2_O_2_-induced neurotoxicity on human neuroblastoma SH-SY5Y cells. Pretreatment of neurons with **119** attenuated the H_2_O_2_ (100 µM)-induced oxidative stress through improving the cell viability, antioxidant status, mitochondrial membrane integrity, and regulation of gene expression [95]. Therefore, it could be utilized as an alternative to some of the toxic synthetic antioxidants and a preventive agent against neurodegeneration [95]. Carmen et al., reported that bikaverin-contaminated products had no negative effect on human health [199]. Kundu et al., also purified **119** from *F*. *oxysporum* f. sp. *ciceris* ITCC-3636 EtOAc extract that had a weak anti-nemic potential towards *M*. *incognita* (LC_50_ 392.9 μg/mL) [88]. Additionally, Son et al., reported that **119** isolated from *F. oxysporum* EF119 showed antimicrobial activities against various phyto-pathogenic oomycetes and fungi. It suppressed the development of tomato late blight by 71% at conc. 300 µg/mL. Therefore, it may be used as a bio-control agent towards *P. infestans*-caused tomato late blight [66].

### 4.9. Terpenoids

Wortmannin (**129**) a steroidal furan, exhibited potent and selective antifungal activity toward *C. albicans* (IC_50_ 0.25 µg/mL and MIC 0.78 µg/mL), compared with amphotericin B (IC_50_ 0.35 and MIC 1.25 µg/mL) [63]. Another study revealed that **129** was a hemorrhagic factor reported from *F. oxysporum* (N17B) that caused different organs hemorrhage and finally death in rats and mice [98,200]. It also showed a powerful inhibitory potential of phosphatidylinositide 3-kinase [201] and had antifungal potential towards *Botrytis allii* [202]. Ergosta-5,8(14),22-trien-7-one,3-hydroxy-(3β,22E) (**130**) was characterized from *F. oxysporum*, which had HCV (hepatitis C virus) NS3 protease inhibitory activity (Ki 99.7 μM/L), compared to VX950 (Ki 3.5 μM/L) in the FRET (fluorescence resonance energy transfer) [99] (Figure 14).

Isoverrucarol (**133**), a trichothecene was isolated from *F. oxysporum* CJS-12, harboring corn produced toxic effects (dose 10 and 20 mg/kg/b.wt., orally) in rats, including body weakness, loss of appetite, stomach severe mucosae, and death. It also caused a definite dermatitis reaction of the epidermis and an edematic-necrotic response of the dermis [100]. FCRR (**134)** a new phytotoxin, having a labdane framework was purified from *F. oxysporum* f. sp. *radicis-lycopersici* (causal agent of *Fusarium* rot and crown rot of tomato). It (conc. 0.25 µg/mL) induced leaf necrosis for Momotaro (a cultivar of tomato, *Lycopersicon esculentum* Mill.) [101]. Chen et al., separated and characterized two novel compounds, fusariumins C (**135**) and D (**136**) from *F. oxysporum* ZZP-R1 derived from *Rumex madaio*. They were assigned as a meroterpene with cyclohexanone unit and a sesquiterpene ester with a conjugated triene and an unusual oxetene ring, respectively based on NMR tools and optical rotation analysis. They had a potent inhibitory effect on *S. aureus* (MICs 6.25 and 25 μM, respectively), however, they were weakly active towards *E. coli* and *C. albicans* in the micro-broth dilution method [102].

The sesquiterpenoid, **137** isolated from *F. oxysporum* LBKURCC41 obtained from *Dahlia variabilis* tubers showed antibacterial activity towards *S. aureus* and *E. coli* (IZD 2.1 mm) in the agar disc diffusion assay [103]. Cosmosporasides F–H (**138**–**140**), new sugar alcohol conjugated acyclic sesquiterpenes isolated from *F. oxysporum* SC0002, showed weak cytotoxic effect towards A549, HepG2, and HeLa (inhibition rates 13–24%) (Figure 15). They exhibited weak antibacterial potential towards *S. aureus*, *B. cereus*, *E. coli*, *S. typhimurium*, and *S. dysenteriae* (growth inhibition rate of 8–21%, conc. 100 µg/mL) in the Alamar blue assay. They also displayed weak inhibition of LPS-induced NO production in RAW 264.7 macrophages (9–16%, conc. 50 µM) [27].

### 4.10. Phenolic and Aromatic Compounds

Podophyllotoxin (**151**), an aryltetralin lignan was reported from *F. oxysporum* isolated from *Juniperus recurva* and quantified by HPLC, LC-MS, and LC-MS/MS [106] (Figure 16). Kılıç et al., reported the isolation of **153** from *F. oxysporum* YP9B that displayed potent cytotoxic potential towards MCF-7, PC-3, and A549 (IC_50_ 15.01, 19.13, and 17.06 µM, respectively), compared to doxorubicin (IC_50_ 0.053, 0.09, 17.75 µM, respectively). It showed antiviral potential towards the HSV type-1 virus that lysed VERO cells. It produced a partial increase in VERO cell viability (conc. 0.312 µM). Moreover, it had a powerful antibacterial potential (MICs 0.47–1.8 µg/mL) towards *B. cereus*, *S. mutans*, *S. aureus*, *E. faecalis*, and *M. smegmatis* [73]. 

### 4.11. Pyran and Furan Derivatives

Chlamydosporol (**158**) a pyran lactone derivative was isolated from marine-mudflat-derived *F. oxysporum* and assessed for its antibacterial potential towards MRSA and MDRSA. It displayed weak antibacterial activity against MRSA and MDRSA (MIC 31.5 µg/mL) [70]. The co-culture of *F. oxysporum* R1 and *A. fumigatus* D afforded neovasinin (**160**) and neovasifuranone B (**163**) that had a weak antimicrobial activity towards *E. coli*, *S. aureus*, and *C. albicans* (MICs ≥ 25 μM) [72] (Figure 17).

### 4.12. Aliphatic Acids

Mixtures of acid esters: **165**–**167**, **168** and **169**, and **170**–**172** were identified from *F. oxysporum* YP9B by NMR, UV, FT-IR, and GC-FID- and LC-QTOF-MS [73]. Only **170**–**172** mixture showed potent cytotoxic activity on MCF-7, PC-3, and A549 (IC_50_ 7.75, 17.75, and 7.51 µM, respectively), compared to doxorubicin (IC_50_ 0.053, 0.09, and 17.75 µM, respectively), where it was about two folds more active than doxorubicin on A549. Only the mixture of **168** and **169** caused a partial cell viability increase (conc. 1.25 µM) in the antiviral assay towards HSV-I. Moreover, they exhibited strong to weak antimicrobial potential towards various tested microorganisms. On the other hand, the mixture of **170**–**172** showed potent activity only against *S. aureus* ATCC25923, *E. faecalis* ATCC29212, *S. mutans* RSKK07038, and *B. cereus* 702 Roma (MIC ranging from 2.1 to 4.3 µg/mL) [73]. Yu et al., purified compounds **173**–**175** from *F. oxysporum* R1 and *A. fumigatus* D co-culture, which displayed weak antimicrobial activity (MICs ≥ 25 μM) towards *E. coli*, *S. aureus*, and *C. albicans* [72] (Figure 18).

### 4.13. Volatile Organic Compounds

GCMS analysis of the VOCs (volatile organic compounds) of *F. oxysporum* isolate 21 obtained from coffee plant rhizosphere, *Meloidogyne exigua* eggs and egg masses revealed the existence of 38 VOCs, five of them were above 1% (dioctyl disulfide, 1-(2-hydroxyethoxy) tridecane or 2-propyldecan-1-ol, 4-methyl-2,6-di-tert-butylphenol, caryophyllene, and acoradiene). VOCs from *F. oxysporum* displayed nematicidal potential towards *M. incognita*, thus it could be useful for the development of bio-control agent for *Meloidogyne* spp. in coffee fields [203].

do Nascimento et al., reported that GCMS of *n*-hexane extract of *F. oxysporum* SS50 isolated from *Smallanthus sonchifolius* revealed twelve compounds; pentadecane, (2*E*,4*E*)-decadienal, hexadecane, octadecane, heptadecane, bis(2-methylpropyl) ester, 1,2-benzenedicarboxylic acid, methyl hexadecanoate or methyl palmitate, (9*Z*,12*Z*)-octadecadienoic acid methyl ester, clionasterol, dehydroergosterol, (9*Z*)-octadecenoic acid methyl ester, and stigmast-4-en-3-one, where fatty acid methyl esters and alkanes were predominated (4.70% (9*Z*)-octadecenoic acid methyl ester, 9.73% methyl hexadecanoate, and 54.45% (9Z,12*Z*)-octadecadienoic acid methyl ester). The *n*-hexane extract possessed cytotoxic activity towards HCT-8, MDA-MB435, and SF295 (% growth inhibition ranging from 83.78 to 97.72%, conc. 50 μg/mL) in the MTT assay. This could be attributed to the mixture of three methyl esters [84].

## 5. Conclusions and Future Research Directions

Currently, more focus has been directed to fungi as they are a wealthy platform for the biosynthesis of a huge number of structural diverse metabolites. *F. oxysporum* is a species with great physiological and morphological variations and its wide-ranging existence in ecological activities worldwide indicates its profoundly diversified and significant role in nature. It can produce various bio-metabolites that may directly and indirectly be utilized as therapeutic agents for various health problems. In this work, 180 metabolites were reported from *F. oxysporum* in the period from 1967 to 2021. Alkaloids quinones, and jasmonates, and anthranilate derivatives represented the major metabolites that were isolated from this fungus (Figure 19).

Although, this big number of reported metabolites, few of them are evaluated for their bioactivities. The assessed activities of these metabolites were antimicrobial, cytotoxicity, nematicidal, antiviral, leishmanicidal, antiviral, and antioxidant. Additionally, there is a lack of pharmacological studies that focus on exploring the possible mechanisms of the active metabolites. In addition, the untested metabolites should be further explored for their possible bioactivities. Co-cultivation experiments should be employed to elicit the production of these metabolites. The discovery of the underlying biosynthetic pathways of these bio-metabolites is needed, which would allow the rational engineering or refactoring of these pathways for industrial purposes. Further, research for identifying the responsible biosynthetic genes for these metabolites may open the opportunity to explore the genetic potential of *F. oxysporum* for discovering novel metabolites by metabolic engineering that could result in more affordable and novel pharmaceutics and food additives. Moreover, studies on the structure-activity relationships and/or derivatization of these fungus metabolites should be carried out.

Although, the reported data revealed that *F. oxysporum* is widely employed for the synthesis of different types of metal nanoparticles that could have various biotechnological, agronomical, pharmaceutical, industrial, and medicinal applications. Many of these biosynthesized NPs possessed favorable antimicrobial potential, especially towards MDR microbes that can be potential alternatives to antibiotics. Further, it was found that the combination of NPs synthesized using *F. oxysporum* with antibiotics produced additive and synergic effects that could represent a new strategy for treating some antibiotics resistant strains and lower the doses of the used antibiotics. *F. oxysporum* might have vast commercial implications in low-cost, room-temperature, ecofriendly syntheses of technologically significant oxide nanomaterials from naturally available potentially cheap raw materials. However, the NPs synthesized from *F. oxysporum* are limited to metals and fewer metal oxides and sulfides. Therefore, future research should focus on developing protocols for implementing the biosynthesis of NPs of other metals, metal oxides, nitrides, and carbides. Research on the toxic effect of these NPs, as well as their effects on animals and human health and accumulation in the environment, is needed.

Additionally, in-vivo studies and clinical trials are needed to elaborate the exact mechanism responsible for their observed bioactivities. There is also a need for evaluating these NPs for their effectiveness towards various diseases, which can open in the future a new avenue in the biomedical field. More research is required for optimizing various reaction conditions to achieve better control over the shape, size, stability, and monodispersity of these NPs. *F. oxysporum* is considered as an efficient enzyme producer. Its enzymes have attracted great interest because of their possible applications in diverse biotechnological and industrial fields such as pharmaceutical, cosmetic, and food industries, organic synthesis, bioremediation, and detoxification of environmental pollutants, delignification, denitrification, or biofuel production. Additionally, they are involved in eco-friendly bioconversion processes of various substrates to highly valuable products that could be preferred more over the chemical synthesis. Research that focuses on engineering enzymes in such a way for maximum stability and activity under appropriate conditions is desirable. Recombinant DNA technology and engineering of proteins are required to improve the industrial production of these enzymes. Additionally, some *F. oxysporum* strains can be utilized as bio-control agents because of their ability to prohibit the growth of several fungal plant pathogens.

## Figures and Tables

**Table 1:** List of compounds isolated from *F. oxysporum* (chemical class, molecular weight and formulae, fungal source, host, and place).

Compound Name	Mol. Wt.	Mol. Formula	Fungal Source	Host (Part, Family)	Place	Ref.
Anthranilic Acid Derivatives						
Dianthramide (**1**)	283	C_16_H_13_NO_4_	*F. oxysporum* f. sp. *dianthi*	*Dianthus caryophyllus*, stem (Caryophyllaceae)	The Netherlands	[56]
Dianthramide B (**2**)	241	C_14_H_11_NO_3_	*F. oxysporum* f. sp. *dianthi*	*Dianthus caryophyllus*, stem (Caryophyllaceae)	The Netherlands	[56]
Hydroxydianthramide B (**3**)	257	C_14_H_11_NO_4_	*F. oxysporum* f. sp. *dianthi*	*Dianthus caryophyllus*, stem (Caryophyllaceae)	The Netherlands	[56]
Hydroxydianthramide M (**4**)	303	C_15_H_15_NO_6_	*F. oxysporum* f. sp. *dianthi*	*Dianthus caryophyllus*, stem (Caryophyllaceae)	The Netherlands	[56]
Hydroxydianthramide S (**5**)	273	C_14_H_11_NO_5_	*F. oxysporum* f. sp. *dianthi*	*Dianthus caryophyllus*, stem (Caryophyllaceae)	The Netherlands	[56]
Hydroxydianthramide R (**6**)	289	C_14_H_11_NO_6_	*F. oxysporum* f. sp. *dianthi*	*Dianthus caryophyllus*, stem (Caryophyllaceae)	The Netherlands	[56]
Methoxydianthramide M (**7**)	317	C_16_H_15_NO_6_	*F. oxysporum* f. sp. *dianthi*	*Dianthus caryophyllus*, stem (Caryophyllaceae)	The Netherlands	[56]
Methoxydianthramide B (**8**)	271	C_15_H_13_NO_4_	*F. oxysporum* f. sp. *dianthi*	*Dianthus caryophyllus*, stem (Caryophyllaceae)	The Netherlands	[56]
Methoxydianthramide S (**9**)	287	C_15_H_13_NO_5_	*F. oxysporum* f. sp. *dianthi*	*Dianthus caryophyllus*, stem (Caryophyllaceae)	The Netherlands	[56]
Methoxydianthramide R (**10**)	303	C_15_H_13_NO_6_	*F. oxysporum* f. sp. *dianthi*	*Dianthus caryophyllus*, stem (Caryophyllaceae)	The Netherlands	[56]
Methoxydianthramide A (**11**)	301	C_16_H_15_NO_5_	*F. oxysporum* f. sp. *dianthi*	*Dianthus caryophyllus*, stem (Caryophyllaceae)	The Netherlands	[56]
Hydroxydianthramide S ethyl ester (**12**)	301	C_16_H_15_NO_5_	*F. oxysporum* f. sp. *dianthi*	*Dianthus caryophyllus*, stem (Caryophyllaceae)	The Netherlands	[56]
Hydroxydianthramide S methyl ester (**13**)	287	C_15_H_13_NO_5_	*F. oxysporum* f. sp. *dianthi*	*Dianthus caryophyllus*, stem (Caryophyllaceae)	The Netherlands	[56]
Methoxydianthramide A methyl ester (**14**)	315	C_15_H_13_NO_5_	*F. oxysporum* f. sp. *dianthi*	*Dianthus caryophyllus*, stem (Caryophyllaceae)	The Netherlands	[56]
Hydroxyanilide B (**15**)	213	C_13_H_11_NO_2_	*F. oxysporum* f. sp. *dianthi*	*Dianthus caryophyllus*, stem (Caryophyllaceae)	The Netherlands	[56]
Hydroxyanilide S (**16**)	229	C_13_H_11_NO_3_	*F. oxysporum* f. sp. *dianthi*	*Dianthus caryophyllus*, stem (Caryophyllaceae)	The Netherlands	[56]
Hydroxyanilide R (**17**)	245	C_15_H_13_NO_5_	*F. oxysporum* f. sp. *dianthi*	*Dianthus caryophyllus*, stem (Caryophyllaceae)	The Netherlands	[56]
Dianthalexin B (**18**)	223	C_14_H_9_NO_2_	*F. oxysporum* f. sp. *dianthi*	*Dianthus caryophyllus*, stem (Caryophyllaceae)	The Netherlands	[56]
Hydroxydianthalexin B (**19**) = Dianthalexin	239	C_14_H_9_NO_3_	*F. oxysporum* f. sp. *dianthi*	*Dianthus caryophyllus*, stem (Caryophyllaceae)	The Netherlands	[56]
Hydroxydianthalexin S (**20**)	255	C_14_H_9_NO_4_	*F. oxysporum* f. sp. *dianthi*	*Dianthus caryophyllus*, stem (Caryophyllaceae)	The Netherlands	[56]
Methoxydianthalexin S (**21**)	269	C_15_H_11_NO_4_	*F. oxysporum* f. sp. *dianthi*	*Dianthus caryophyllus*, stem (Caryophyllaceae)	The Netherlands	[56]
**Fumonisins**						
Fumonisin B_1_ (**22**)	721	C_34_H_59_NO_15_	*F. oxysporum*	*Asparagus officinalis* (Asparagaceae)	Western Poland	[57]
Hydroxylated fumonisin C_1_ (**23**)	723	C_33_H_57_NO_16_	*F. oxysporum* CAR	*Dianthus caryophyllus* (Caryophyllaceae)	Taejon, Korea	[58,59]
Fumonisin C_1_ (**24**)	707	C_33_H_57_NO_15_	*F. oxysporum* CAR	*Dianthus caryophyllus* (Caryophyllaceae)	Taejon, Korea	[58,59]
Fumonisin C_3_ (**25**)	691	C_33_H_57_NO_14_	*F. oxysporum* CAR	*Dianthus caryophyllus* (Caryophyllaceae)	Taejon, Korea	[58,59]
Fumonisin C_4_ (**26**)	675	C_33_H_57_NO_13_	*F. oxysporum* CAR	*Dianthus caryophyllus* (Caryophyllaceae)	Taejon, Korea	[58,59]
*Iso*-Fumonisin C_1_ (**27**)	707	C_33_H_57_NO_15_	*F. oxysporum* KCTC 16654	*Asparagus officinalis* (Asparagaceae)	Taejon, Korea	[60]
*N*-Acetylated OH-fumonisin C_1_ (**28**)	765	C_35_H_59_NO_17_	*F. oxysporum* KCTC 16654	*Asparagus officinalis* (Asparagaceae)	Taejon, Korea	[60]
*N*-Acetylated fumonisin C_1_ (**29**)	749	C_35_H_59_NO_16_	*F. oxysporum* KCTC 16654	*Asparagus officinalis* (Asparagaceae)	Taejon, Korea	[60]
*N*-Acetylated *iso*-fumonisin C_1_ (**30**)	749	C_35_H_59_NO_16_	*F. oxysporum* KCTC 16654	*Asparagus officinalis* (Asparagaceae)	Taejon, Korea	[60]
**Jasmonates derivatives**						
(−)-Jasmonic acid (**31**)	210	C_12_H_18_O_3_	*F. oxysporum* f. sp. *matthiolae*	Cultured	The Netherlands	[61]
(+)-7-*Iso*-jasmonic acid (**32**)	210	C_12_H_18_O_3_	*F. oxysporum* f. sp. *matthiolae*	Cultured	The Netherlands	[61]
(1*S*,2*R*)-3-Oxo-2-(2*Z*-pentenyl) cyclopentane-1-butyric acid (**33**)	238	C_14_H_22_O_3_	*F. oxysporum* f. sp. *matthiolae*	Cultured	The Netherlands	[61]
(1*S*,2*S*)-3-(1*S*,2*S*)-3-Oxo-2-(2*Z*-pentenyl)cyclopentane-1-butyric acid (**34**)	238	C_14_H_22_O_3_	*F. oxysporum* f. sp. *matthiolae*	Cultured	The Netherlands	[61]
(1*S*,2*R*)-3-Oxo-2-(2*Z*-pentenyl) cyclopentane-1-hexanoic acid (**35**)	266	C_16_H_26_O_3_	*F. oxysporum* f. sp. *matthiolae*	Cultured	The Netherlands	[61]
(1*S*,2*S*)-3-Oxo-2-(2*Z*-pentenyl) cyclopentane-1-hexanoic acid (**36**)	266	C_16_H_26_O_3_	*F. oxysporum* f. sp. *matthiolae*	Cultured	The Netherlands	[61]
(1*S*,2*R*)-3-Oxo-2-(2*Z*-pentenyl) cyclopentane-1-octanoic acid (**37**)	294	C_18_H_30_O_3_	*F. oxysporum* f. sp. *matthiolae*	Cultured	The Netherlands	[61]
(1*S*,2*S*)-3-Oxo-2-(2*Z*-pentenyl) cyclopentane-1-octanoic acid (**38**)	294	C_18_H_30_O_3_	*F. oxysporum* f. sp. *matthiolae*	Cultured	The Netherlands	[61]
9,10-Dihydrojasmonic acid (**39**)	212	C_12_H_20_O_3_	*F. oxysporum* f. sp. *matthiolae*	Cultured	The Netherlands	[61]
9,10-Dihydro-7-*iso*-jasmonic acid (**40**)	212	C_12_H_20_O_3_	*F. oxysporum* f. sp. *matthiolae*	Cultured	The Netherlands	[61]
(1*S*,2*R*)-3-Oxo-2-pentylcyclopentane-1-butyric acid (**41**)	240	C_14_H_24_O_3_	*F. oxysporum* f. sp. *matthiolae*	Cultured	The Netherlands	[61]
(1*S*,2*S*)-3-Oxo-2-pentylcyclopentane-1-butyric acid (**42**)	240	C_14_H_24_O_3_	*F. oxysporum* f. sp. *matthiolae*	Cultured	The Netherlands	[61]
(1*S*,2*R*)-3-Oxo-2-pentylcyclopentane-1-hexanoic acid (**43**)	268	C_16_H_28_O_3_	*F. oxysporum* f. sp. *matthiolae*	Cultured	The Netherlands	[61]
(1*S*,2*S*)-3-Oxo-2-pentylcyclopentane-1-hexanoic acid (**44**)	268	C_16_H_28_O_3_	*F. oxysporum* f. sp. *matthiolae*	Cultured	The Netherlands	[61]
(1*S*,2*R*)-3-Oxo-2-pentylcyclopentane-1-octanoic acid (**45**)	296	C_18_H_32_O_3_	*F. oxysporum* f. sp. *matthiolae*	Cultured	The Netherlands	[61]
(1*S*,2*S*)-3-Oxo-2-pentylcyclopentane-1-octanoic acid (**46**)	296	C_18_H_32_O_3_	*F. oxysporum* f. sp. *matthiolae*	Cultured	The Netherlands	[61]
7-*Iso*-cucurbic acid (**47**)	212	C_12_H_20_O_3_	*F. oxysporum* f. sp. *matthiolae*	Cultured	The Netherlands	[61]
Cucurbic acid (**48**)	212	C_12_H_20_O_3_	*F. oxysporum* f. sp. *matthiolae*	Cultured	The Netherlands	[61]
N-(−)-Jasmonoyl-(S)-isoleucine (**49**)	323	C_18_H_29_NO_4_	*F. oxysporum* f. sp. *matthiolae*	Cultured	The Netherlands	[61]
N-(+)-7-*Iso*-jasmonoyl-(S)-isoleucine (**50**)	323	C_18_H_29_NO_4_	*F. oxysporum* f. sp. *matthiolae*	Cultured	The Netherlands	[61]
N-(9,10-Dihydrojasmonoyl)-(*S*)-isoleucine (**51**)	325	C_18_H_31_NO_4_	*F. oxysporum* f. sp. *matthiolae*	Cultured	The Netherlands	[61]
N-(9,10-Dihydro-7-*iso*-jasmonoyl)-(*S*)-isoleucine (**52**)	325	C_18_H_31_NO_4_	*F. oxysporum* f. sp. *matthiolae*	Cultured	The Netherlands	[61]
**Alkaloids**						
Oxysporidinone (**53**)	489	C_28_H_43_NO_6_	*F. oxysporum* CBS 330.95	Plant	Sweden	[62]
(−)-Oxysporidinone (**54**)	489	C_28_H_43_NO_6_	*F. oxysporum* N17B	Cultured	Mississippi, USA	[63]
			*F. oxysporum* EPH2R_AA_	*Ephedra fasciculate*, root (Ephedraceae)	Sonoran Desert, USA	[64]
(−)-4’-Hydroxyl oxysporidinone (**55**)	491	C_28_H_45_NO_6_	*F. oxysporum*	*Cinnamomum kanehirae*, bark (Lauraceae)	Jiaoban Mountain, Taiwan. China	[65]
(−)-6-Deoxyoxysporidinone (**56**)	473	C_28_H_43_NO_5_	*F. oxysporum* EPH2R_AA_	*Ephedra fasciculate*, root (Ephedraceae)	Sonoran Desert, USA	[64]
(−)-4,6’-Anhydrooxysporidinone (**57**)	471	C_28_H_41_NO_5_	*F. oxysporum* EPH2R_AA_	*Ephedra fasciculate*, root (Ephedraceae)	Sonoran Desert, USA	[64]
			*F. oxysporum*	*Cinnamomum kanehirae*, bark (Lauraceae)	Jiaoban Mountain, Taiwan. China	[65]
(−)-Sambutoxin (**58**)	453	C_28_H_39_NO_5_	*F. oxysporum* N17B	Cultured	Mississippi, USA	[63]
6-*Epi*-oxysporidinone (**59**)	489	C_28_H_43_NO_6_	*F. oxysporum* N17B	Cultured	Mississippi, USA	[63]
Dimethyl ketal of oxysporidinone (**60**)	535	C_30_H_49_NO_7_	*F. oxysporum* N17B	Cultured	Mississippi, USA	[63]
*N*-Demethylsambutoxin (**61**)	439	C_27_H_37_NO_4_	*F. oxysporum* N17B	Cultured	Mississippi, USA	[63]
2-Phenylpropionyl-2-piperidinone-3-(*R*)-yl ester (**62**)	247	C_14_H_17_NO_3_	*F. oxysporum*	*Cinnamomum kanehirae*, bark (Lauraceae)	Jiaoban Mountain, Taiwan. China	[65]
Fusaric acid (**63**)	179	C_10_H_13_NO_2_	*F. oxysporum* EF119	*Capsicum annuum*, root (Solanaceae)	Taejon, Korea	[66]
			*F. oxysporum*	*Vitis vinifera*, plant (Vitaceae)	Firenze, Italy	[67]
9,10-Dehydrofusaric acid (**64**)	177	C_10_H_11_NO_2_	*F. oxysporum* f. sp. *pisi*, F42 and F69	Cultured	USA	[68]
Fusaricate A (**65**)	253	C_13_H_19_NO_4_	*F. oxysporum*	*Drepanocarpus lunatus*, fruit (Fabaceae)	Douala, Cameroon	[69]
Fusaricate B (**66**)	269	C_13_H_19_NO_5_	*F. oxysporum*	*Drepanocarpus lunatus*, fruit (Fabaceae)	Douala, Cameroon	[69]
Fusaricate C (**67**)	283	C_14_H_21_NO_5_	*F. oxysporum*	*Drepanocarpus lunatus*, fruit (Fabaceae)	Douala, Cameroon	[69]
Fusaricate D (**68**)	313	C_15_H_23_NO_6_	*F. oxysporum*	*Drepanocarpus lunatus*, fruit (Fabaceae)	Douala, Cameroon	[69]
Fusaricate E (**69**)	313	C_15_H_23_NO_6_	*F. oxysporum*	*Drepanocarpus lunatus*, fruit (Fabaceae)	Douala, Cameroon	[69]
Fusaricate F (**70**)	311	C_15_H_21_NO_6_	*F. oxysporum*	*Drepanocarpus lunatus*, fruit (Fabaceae)	Douala, Cameroon	[69]
Fusaricate G (**71**)	311	C_15_H_21_NO_6_	*F. oxysporum*	*Drepanocarpus lunatus*, fruit (Fabaceae)	Douala, Cameroon	[69]
10-Hydroxy-11-chlorofusaric acid (**72**)	215	C_9_H_10_ClNO_3_	*F. oxysporum*	*Drepanocarpus lunatus*, fruit (Fabaceae)	Douala, Cameroon	[69]
Oxysporizoline (**73**)	446	C_28_H_22_N_4_O_2_	*F. oxysporum*	Marine mudflat	Suncheon Bay, Korea	[70]
1*H*-Indol-3-butanamide (**74**)	202	C_12_H_14_N_2_O	*F. oxysporum*	Marine mudflat	Suncheon Bay, Korea	[70]
Butenolide (**75**)	141	C_6_H_7_NO_3_	*F. oxysporum*	Marine mudflat	Suncheon Bay, Korea	[70]
*Cyclo*-(L-prolyl-L-glycine) (**76**)	154	C_7_H_10_N_2_O_2_	*F. oxysporum* UDLAP 21-92	Cultured	Mexico	[71]
*Cyclo*-(L-prolyl-L-valine) (**77**)	196	C_10_H_16_N_2_O_2_	*F. oxysporum* UDLAP 21-92	Cultured	Mexico	[71]
*Cyclo*-(L-leucyl-L-proline) (**78**)	210	C_11_H_18_N_2_O_2_	*F. oxysporum* UDLAP 21-92	Cultured	Mexico	[71]
(*S*,*E*)-Methyl-2-(2,4-dimethylhex-2-enamido)acetate (**79**)	213	C_11_H_19_NO_3_	*F. oxysporum* R1 and *Aspergillus fumigatus* D co-culture	*Rumex madaio* (Polygonaceae) *Edgeworthia chrysantha* (Thymelaeaceae)	China	[72]
2-Oxo-8-azatricyclo [9.3.1.1^3,7^]-hexadeca -1(15),3(16),4,6,11,13-hexaen-10-one (**80**)	225	C_14_H_11_NO_2_	*F. oxysporum* YP9B	*Solanum lycopersicum*, root, (Solanaceae)	Pazar-Rize, Turkey	[73]
Fusarioxazin (**81**)	337	C_20_H_19_NO_4_	*F. oxysporum*	*Vicia faba*, root, (Fabaceae)	Assiut, Egypt	[18]
*Epi*-trichosetin (**82**)	359	C_21_H_29_NO_4_	*F. oxysporum* FKI-4553	Cultured	Japan	[74]
Trichosetin (**83**)	359	C_21_H_29_NO_4_	*F. oxysporum* FKI-4553	Cultured	Japan	[74]
*N*-(2-Phenylethyl)acetamide (**84**)	177	C_11_H_15_NO	*F. oxysporum* R1 and *Aspergillus fumigatus* D co-culture	*Rumex madaio* (Polygonaceae) *Edgeworthia chrysantha* (Thymelaeaceae)	China	[72]
Siderophore (**85**)	344	C_16_H_12_N_2_O_7_	*F. oxysporum*	*Musa acuminata* (Musaceae)	Colombo, Sri Lanka	[75]
Vinblastine (**86**)	810	C_46_H_58_N_4_O_9_	*F. oxysporum*	*Catharanthus roseus*, leaf (Apocynaceae)	India	[76]
Vincristine (**87**)	824	C_46_H_58_N_4_O_10_	*F. oxysporum*	*Catharanthus roseus*, leaf (Apocynaceae)	India	[76]
Taxol (**88**)	853	C_47_H_51_NO_14_	*F. oxysporum*	*Rhizophora annamalayana*, leaf (*Rhizophoraceae)*	Vellar Estuary, Tamil Nadu	[77]
Flavin adenine dinucleotide (**89**)	785	C_27_H_33_N_9_O_15_P_2_	*F. oxysporum*	Cultured	USA	[78]
Flavin adenine dinucleotide-N(5)-Nitrobutane (**90**)	888	C_31_H_42_N_10_O_17_P_2_	*F. oxysporum* ATCC 695	Cultured	USA	[78]
			*F. oxysporum* ATCC 695	Cultured	USA	[79]
**Cyclic depsipeptides**						
Enniatin A (**91**)	681	C_36_H_63_N_3_O_9_	*F. oxysporum* f. sp. *pisi*	Cultured	Germany	[80]
Enniatin A1 (**92**)	667	C_35_H_61_N_3_O_9_	*F. oxysporum f. sp. melonis* ITEM 3464	*Cucumis melo* (Cucurbitaceae)	Rome, Italy	[81]
			*F. oxysporum* N17B	Cultured	Mississippi, USA	[63]
Enniatin B (**93**)	639	C_33_H_57_N_3_O_9_	*F. oxysporum* f. sp. *pisi*	Cultured	Germany	[80]
			*F. oxysporum f. sp. melonis* ITEM 3464	*Cucumis melo* (Cucurbitaceae)	Rome, Italy	[81]
Enniatin B1 (**94**)	653	C_34_H_59_N_3_O_9_	*F. oxysporum f. sp. melonis* ITEM 3464	*Cucumis melo* (Cucurbitaceae)	Rome, Italy	[81]
			*F. oxysporum* N17B	Cultured	Mississippi, USA	[63]
Enniatin C (**95**)	681	C_36_H_63_N_3_O_9_	*F. oxysporum* f. sp. *pisi*	Cultured	Germany	[80]
Enniatin H (**96**)	653	C_34_H_59_N_3_O_9_	*F. oxysporum* FB1501 (KFCC 11363P)	Soil	Korea	[82]
			*F. oxysporum* KFCC 11363P	Soil	Korea	[83]
Enniatin I (**97**)	667	C_35_H_61_N_3_O_9_	*F. oxysporum* FB1501 (KFCC 11363P)	Soil	Korea	[82]
			*F. oxysporum* KFCC 11363P	Soil	Korea	[83]
Enniatin MK 1688 (**98**)	681	C_36_H_63_N_3_O_9_	*F. oxysporum* FB1501 (KFCC 11363P)	Soil	Korea	[82]
			*F. oxysporum KFCC 11363P*	Soil	Korea	[83]
Beauvericin (**99**)	783	C_45_H_57_N_3_O_9_	*F. oxysporum* f. sp. *melonis* ITEM 3464	*Cucumis melo* (Cucurbitaceae)	Rome, Italy	[81]
			*F. oxysporum* FB1501 (KFCC 11363P)	Soil	Korea	[82]
			*F. oxysporum* EPH2R_AA_	*Ephedra fasciculate*, root (Ephedraceae)	Sonoran Desert, USA	[64]
			*F. oxysporum* KFCC 11363P	Soil	Korea	[83]
			*F. oxysporum*	*Cinnamomum kanehirae*, bark (Lauraceae)	Jiaoban Mountain, Taiwan. China	[65]
			*F. oxysporum* SS46 and SS50	*Smallanthus sonchifolius*, root (Asteraceae)	Brazil	[84]
			*F. oxysporum*	*Drepanocarpus lunatus*, fruit (Fabaceae)	Douala, Cameroon	[69]
			*F. oxysporum* LCP 531	Soil	France	[85]
**Cyclic peptides**						
Cyclosporine A (**100**)	1201	C_62_H_111_N_11_O_12_	*F. oxysporum* S6	Soil of soybean field	Salto, Buenos Aires, Argentina	[86]
**Glucosylceramides**						
Fusaruside (**101**)	751	C_43_H_77_NO_9_	*F. oxysporum*	*Cinnamomum kanehirae*, bark (Lauraceae)	Jiaoban Mountain, Taiwan, China	[65]
(2S,2′*R*,3*R*,3′*E*,4*E*,8*E*)-1-*O*-*D*-Glucopyranosyl-2-*N*-(2′-hydroxy-3′-octadecenoyl)-3-hydroxy-9-methyl-4,8- sphingadienine (**102**)	753	C_43_H_79_NO_9_	*F. oxysporum*	*Cinnamomum kanehirae*, bark (Lauraceae)	Jiaoban Mountain, Taiwan, China	[65]
			*F. oxysporum* IOC 4247	Cultured	Brazil	[87]
N-2′-Hydroxyoctadecanoic-1-*β*-*D*-glucopyranosyl-9-methy-l-4-hydroxy-4,8- sphingadienine (**103**)	771	C_43_H_81_NO_10_	*F. oxysporum* IOC 4247	Cultured	Brazil	[87]
N-2′-Hydroxyeicosanoyl- 1-*β*-*D*-glucopyranosyl-9-methyl-4,8- sphingadienine (**104**)	783	C_45_H_85_NO_9_	*F. oxysporum* IOC 4247	Cultured	Brazil	[87]
**Naphthoquinone derivatives**						
Anhydrofusarubin (**105**)	288	C_15_H_12_O_6_	*F. oxysporum* SS46 and SS50	*Smallanthus sonchifolius*, root (Asteraceae)	Brazil	[84]
			*F. oxysporum* f. sp. *ciceris* ITCC 3636	Cultured	India	[88]
8-*O*-Methylbostrycoidin (**106**)	299	C_16_H_13_NO_5_	*F. oxysporum*	*Citrus sinensis*, root (Rutaceae)	Florida, USA	[89]
			*F. oxysporum*	*Citrus sinensis*, root (Rutaceae)	Florida, USA	[90]
Fusarubin (**107**)	306	C_15_H_14_O_7_	*F. oxysporum* f. sp. *ciceris* ITCC 3636	Cultured	India	[88]
9-*O*-Methylfusarubin (**108**) = 8-*O*-Methylfusarubin	320	C_16_H_16_O_7_	*F. oxysporum*	*Citrus sinensis*, root (Rutaceae)	Florida, USA	[89]
			*F. oxysporum*	*Citrus sinensis*, root (Rutaceae)	Florida, USA	[90]
			*F. oxysporum* f. sp. *ciceris* ITCC 3636	Cultured	India	[88]
3-*O*-Methyl-9-*O*-methylfusarubin (**109**) = 3-*O*-Methyl-8-*O*-methylfusarubin	334	C_17_H_18_O_7_	*F. oxysporum*	*Citrus sinensis*, root (Rutaceae)	Florida, USA	[89]
			*F. oxysporum* f. sp. *ciceris* ITCC 3636	Cultured	India	[88]
9-*O*-Methylanhydrofusarubin (**110**)	302	C_16_H_14_O_6_	*F. oxysporum*	*Citrus sinensis*, root (Rutaceae)	Florida, USA	[89]
8-*O*-Methyljavanicin (**111**)	304	C_16_H_16_O_6_	*F. oxysporum*	*Citrus sinensis*, root (Rutaceae)	Florida, USA	[89]
			*F. oxysporum*	*Citrus sinensis*, root (Rutaceae)	Florida, USA	[90]
8-*O*-Methylsolaniol (**112**)	306	C_16_H_18_O_6_	*F. oxysporum*	*Citrus sinensis*, root (Rutaceae)	Florida, USA	[89]
			*F. oxysporum*	*Citrus sinensis*, root (Rutaceae)	Florida, USA	[90]
8-*O*-Methyl-2-hydroxyjavanicin (**113**)	306	C_15_H_14_O_7_	*F. oxysporum*	*Citrus sinensis*, root (Rutaceae)	Florida, USA	[89]
			*F. oxysporum*	*Citrus sinensis*, root (Rutaceae)	Florida, USA	[90]
Nectriafurone (**114**)	304	C_15_H_12_O_7_	*F. oxysporum*	*Citrus sinensis*, root (Rutaceae)	Florida, USA	[91]
			*F. oxysporum*	*Citrus sinensis*, root (Rutaceae)	Florida, USA	[90]
Nectriafurone-8-methy ether (**115**)	318	C_16_H_14_O_7_	*F. oxysporum*	*Citrus sinensis*, root (Rutaceae)	Florida, USA	[91]
**Anthraquinone derivatives**						
Rhodolamprometrin (**116**)	314	C_16_H_10_O_7_	*F. oxysporum* *CECIS*	*Ephedra fasciculate*, root (Ephedraceae)	Sonoran Desert, USA	[64]
2-Acetyl-3,8-dihydroxy-6-methoxy-anthraquinone (**117**)	312	C_17_H_12_O_6_	*F. oxysporum*	*Citrus sinensis*, root (Rutaceae)	Florida, USA	[92]
2-(1-Hydroxyethyl)-3,8-dihydroxy-6-methoxy-anthraquinone (**118**)	314	C_17_H_14_O_6_	*F. oxysporum*	*Citrus sinensis*, root (Rutaceae)	Florida, USA	[92]
**Xanthone derivatives**						
Bikaverin (**119**)	382	C_20_H_14_O_8_	*F. oxysporum*	*Cultured*	USA	[93]
			*F. oxysporum* CECIS	*Ephedra fasciculate*, root (Ephedraceae)	Sonoran Desert, USA	[64]
			*F. oxysporum*	Cultured	USA	[94]
			*F. oxysporum* EF119	*Capsicum annuum*, root (Solanaceae)	Taejon, Korea	[66]
			*F. oxysporum* f. sp. lycopersici	Rhizosphere soil of tomato plant	India	[95]
			*F. oxysporum* f. sp. *ciceris* ITCC 3636	Cultured	India	[88]
			*F. oxysporum* LCP 531	Soil	France	[85,96]
Norbikaverin (**120**)	352	C_19_H_12_O_7_	*F. oxysporum* LCP 531	Soil	France	[85,96]
Oxo-Pre-bikaverin (**121**)	338	C_18_H_10_O_7_	*F. oxysporum* LCP 531	Soil	France	[85]
Me-oxo-pre-bikaverin (**122**)	352	C_19_H_12_O_7_	*F. oxysporum* LCP 531	Soil	France	[85]
Dinor-bikaverin (**123**)	354	C_18_H_10_O_8_	*F. oxysporum* LCP 531	Soil	France	[85]
Pre-bikaverin (**124**)	324	C_18_H_12_O_6_	*F. oxysporum* LCP 531	Soil	France	[85]
6-Deoxybikaverin (**125**)	366	C_20_H_14_O_7_	*F. oxysporum* *CECIS*	*Ephedra fasciculate*, root (Ephedraceae)	Sonoran Desert, USA	[64]
**Terpenoids**						
Ergosterol (**126**)	396	C_28_H_44_O	*Fusarium oxysporum* CM 192679	Cultured	Kew, England	[97]
			*F. oxysporum* LCP 531	Soil	France	[85]
Ergosterol peroxide (**127**)	428	C_28_H_44_O_3_	*F. oxysporum* CM 192679	Cultured	Kew, England	[97]
Cerevisterol (**128**)	430	C_28_H_46_O_3_	*F. oxysporum* CM 192679	Cultured	Kew, England	[97]
			*F. oxysporum*	*Cinnamomum kanehirae*, bark (Lauraceae)	Jiaoban Mountain, Taiwan, China	[65]
			*F. oxysporum*	*Vicia faba*, root (Fabaceae)	Assiut, Egypt	[18]
Wortmannin (**129**)	428	C_23_H_24_O_8_	*F. oxysporum* N17B	Soil sample	Grassy field near the city of Lakselv in the Arctic region of Norway	[98]
			*F. oxysporum* N17B	Cultured	Mississippi, USA	[63]
H1-A = Ergosta-5,8 (14),22-trien-7-one, 3-hydroxy-(3*β*, 22E) (**130**)	410	C_28_H_42_O_2_	*F. oxysporum* *SIPI-4004*	Cultured	China	[99]
(22*E*,24*R*)-Stigmasta-5,7,22-trien-3-*β*-ol (**131**)	410	C_29_H_46_O	*F. oxysporum*	*Vicia faba*, root (Fabaceae)	Assiut, Egypt	[18]
Stigmasta-4,6,8(14),22-tetraen-3-one (**132**)	406	C_29_H_42_O	*F. oxysporum*	*Vicia faba*, root (Fabaceae)	Assiut, Egypt	[18]
Isoverrucarol *(***133**)	266	C_15_H_22_O_4_	*Fusarium oxysporum* CJS-12	*Zea mays* (Poaceae)	Jeongsun District, Kangwon, South Korea	[100]
FCRR-toxin (**134**)	346	C_20_H_26_O_5_	*F. oxysporum* f. sp. *radicis-lycopersici*	*Lycopersicon esculentum*, root (Solanaceae)	Japan	[101]
Fusariumin C (**135**)	332	C_21_H_32_O_3_	*F. oxysporum* ZZP-R1	*Rumex madaio* (Polygonaceae)	Putuo Island, Zhoushan, China	[102]
Fusariumin D (**136**)	264	C_16_H_24_O_3_	*F. oxysporum* ZZP-R1	*Rumex madaio* (Polygonaceae)	Putuo Island, Zhoushan, China	[102]
(1*R*,2*S*,3*R*)-3-((*S*)-2-Hydroxy-6-methylhept-5-en-2-yl)-1,2- dimethylcyclopentanol (**137**)	240	C_15_H_28_O_2_	*F. oxysporum* LBKURCC41	*Dahlia variabilis*, tuber (Asteraceae)	Padang Luar, West Sumatra, Indonesia	[103]
Cosmosporaside F (**138**)	536	C_26_H_48_O_11_	*F. oxysporum* SC0002	Soil	Dinghu Mountain Biosphere Reserve, Guangdong, China	[27]
Cosmosporaside G (**139**)	532	C_27_H_48_O_10_	*F. oxysporum* SC0002	Soil	Dinghu Mountain Biosphere Reserve, Guangdong, China	[27]
Cosmosporaside H (**140**)	546	C_28_H_50_O_10_	*F. oxysporum* SC0002	Soil	Dinghu Mountain Biosphere Reserve, Guangdong, China	[27]
Ginkgolide B (**141**)	424	C_20_H_24_O_10_	*F. oxysporum* *SYP0056*	*Ginkgo biloba*, root bark *(Ginkgoaceae)*	Forest site, Changbai Mountain, China	[104]
Trichothecenes T-2 toxin (**142**)	466	C_24_H_34_O_9_	*F. oxysporum* 598	*Baccharis* spp. (Asteraceae)	Brazil	[105]
Trichothecenes HT-2 toxin (**143**)	424	C_22_H_32_O_8_	*F. oxysporum* 598	*Baccharis* spp. (Asteraceae)	Brazil	[105]
3′-OH T-2 toxin (**144**)	482	C_24_H_34_O_10_	*F. oxysporum* 598	*Baccharis* spp. (Asteraceae)	Brazil	[105]
Diacetoxyscirpenol (**145**)	366	C_19_H_26_O_7_	*F. oxysporum* 598	*Baccharis* spp. (Asteraceae)	Brazil	[105]
**Phenolic and aromatics compounds**						
6henolic and aromati (**146**)	300	C_16_H_12_O_6_	*F. oxysporum* f. sp. *pisi*	Cultured	The Netherlands	[106]
6α-Hydroxymaackiainisoflavan (**147**)	302	C_16_H_14_O_6_	*F. oxysporum* f. sp. *pisi*	Cultured	The Netherlands	[106]
Pestalotiollide A (**148**)	386	C_21_H_22_O_7_	*F. oxysporum*	*Drepanocarpus lunatus*, fruit (Fabaceae)	Douala, Cameroon	[69]
Pestalotiollide B (**149**)	386	C_21_H_22_O_7_	*F. oxysporum*	*Drepanocarpus lunatus*, fruit (Fabaceae)	Douala, Cameroon	[69]
Dehydroisopenicillide (**150**)	370	C_21_H_22_O_6_	*F. oxysporum*	*Drepanocarpus lunatus*, fruit (Fabaceae)	Douala, Cameroon	[69]
Podophyllotoxin (**151**)	414	C_22_H_22_O_8_	*F. oxysporum*	*Juniperus recurva*, (Cupressaceae)	Gulmarg Region, South Kashmir, India	[107]
*P*-Hydroxyphenylacetate (**152**)	152	C_8_H_8_O_3_	*F. oxysporum*	Cultured	Hungary	[108]
(1-Benzyl-2-methoxy-2-oxoethyl)-2-hydroxy-3-methylbutanoate (**153**)	280	C_15_H_20_O_5_	*F. oxysporum* YP9B	*Solanum lycopersicum*, root (Solanaceae)	Pazar-Rize, Turkey	[73]
Styrene (**154**)	104	C_8_H_8_	*F. oxysporum* 13-8	*Prunus dulcis*, hull (Rosaceae)	California, USA	[109]
1-Ethyl-4-methoxybenzene (**155**)	136	C_9_H_12_O	*F. oxysporum* 13-8	*Prunus dulcis*, hull (Rosaceae)	California, USA	[109]
Phenylacetic acid (**156**)	136	C_8_H_8_O_2_	*F. oxysporum*	Cultured	Hungary	[108]
(+)-Fusarinolic acid (**157**)	194	C_11_H_14_O_3_	*F. oxysporum*	*Cinnamomum kanehirae*, bark (Lauraceae)	Jiaoban Mountain, Taiwan. China	[65]
**Pyran and furan derivatives**						
Chlamydosporol (**158**)	226	C_11_H_14_O_5_	*F. oxysporum*	Marine mudflat	Suncheon Bay, Korea	[70]
Gibepyrone D (**159**)	194	C_10_H_10_O_4_	*F. oxysporum*	*Cinnamomum kanehirae*, bark (Lauraceae)	Jiaoban Mountain, Taiwan. China	[65]
Neovasinin (**160**)	308	C_17_H_24_O_5_	*F. oxysporum* R1 and *Aspergillus fumigatus* D co-culture	-*Rumex madaio* (Polygonaceae) -*Edgeworthia chrysantha* (Thymelaeaceae)	China	[72]
Oxysporone (**161**)	156	C_7_H_8_O_4_	*Fusarium oxysporum*	Cultured	England	[110]
Dihydrofuran-2(3H)-one (**162**)	86	C_4_H_6_O_2_	*F. oxysporum* 13-8	*Prunus dulcis*, hull (Rosaceae)	California, USA	[109]
Neovasifuranone B (**163**)	282	C_16_H_26_O_4_	*F. oxysporum* R1 and *Aspergillus fumigatus* D co-culture	-*Rumex madaio* (Polygonaceae) -*Edgeworthia chrysantha* (Thymelaeaceae)	China	[72]
2-Pentylfuran (**164**)	138	C_9_H_14_O	*F. oxysporum* 13-8	*Prunus dulcis*, hull (Rosaceae)	California, USA	[109]
**Aliphatic acids**						
2,3-Dihydroxypropanoic hexadecanoic anhydride (**165**)	344	C_19_H_36_O_5_	*F. oxysporum* YP9B	*Solanum lycopersicum*, root (Solanaceae)	Pazar-Rize, Turkey	[73]
2,3-Dihydroxypropanoic (9*Z*)-octadecenoic anhydride (Δ^9^;*Z*) (**166**)	370	C_21_H_38_O_5_	*F. oxysporum* YP9B	*Solanum lycopersicum*, root (Solanaceae)	Pazar-Rize, Turkey	[73]
2,3-Dihydroxypropanoic (9*Z*,12*Z*)-octadecadienoic anhydride (Δ^9,12^;*Z*) (**167**)	368	C_21_H_36_O_5_	*F. oxysporum* YP9B	*Solanum lycopersicum*, root (Solanaceae)	Pazar-Rize, Turkey	[73]
2,3-Dihydroxypropanoic (11*Z*)-octadecenoic anhydride (Δ^11^;*Z*) (**168**)	370	C_21_H_38_O_5_	*F. oxysporum* YP9B	*Solanum lycopersicum*, root (Solanaceae)	Pazar-Rize, Turkey	[73]
2,3-Dihydroxypropanoic (9*E*,12*E*)-octadecadienoic anhydride (Δ^9,12^;*E*) (**169**)	368	C_21_H_36_O_5_	*F. oxysporum* YP9B	*Solanum lycopersicum*, root (Solanaceae)	Pazar-Rize, Turkey	[73]
3-Hydroxy-1,2,6,10-tetramethylundecyl hexzadecanoate (**170**)	482	C_31_H_62_O_3_	*F. oxysporum* YP9B	*Solanum lycopersicum*, root (Solanaceae)	Pazar-Rize, Turkey	[73]
3-Hydroxy-1,2,6,10-tetramethylundecyl -octadecaenoate (Δ^9^;*E*) (**171**)	508	C_33_H_64_O_3_	*F. oxysporum* YP9B	*Solanum lycopersicum*, root (Solanaceae)	Pazar-Rize, Turkey	[73]
3-Hydroxy-1,2,6,10-tetramethylundecyl octadecanoate (**172**)	510	C_33_H_66_O_3_	*F. oxysporum* YP9B	*Solanum lycopersicum*, root (Solanaceae)	Pazar-Rize, Turkey	[73]
*α*-Linolenic acid (**173**)	278	C_18_H_30_O_2_	*F. oxysporum* R1 and *Aspergillus fumigatus* D co-culture	-*Rumex madaio* (Polygonaceae) -*Edgeworthia chrysantha* (Thymelaeaceae)	China	[72]
*α*-Elaeostearic acid *(***174**)	278	C_18_H_30_O_2_	*F. oxysporum* R1 and *Aspergillus fumigatus* D co-culture	-*Rumex madaio* (Polygonaceae) -*Edgeworthia chrysantha* (Thymelaeaceae)	China	[72]
Palmitoleic acid (**175**)	254	C_16_H_30_O_2_	*F. oxysporum* R1 and *Aspergillus fumigatus* D co-culture	-*Rumex madaio* (Polygonaceae) -*Edgeworthia chrysantha* (Thymelaeaceae)	China	[72]
**Sugar derivatives**						
*α*-*D*-Mannopyranosyl-(1→2)-*α*-*D*-glucopyranosyl (**176**)	342	C_12_H_22_O_11_	*F. oxysporum* L.	Cultured	France	[111]
*α*-*D*-Mannopyranosyl-(1→2)-*β*-*D*-glucopyranosyl (**177**)	342	C_12_H_22_O_11_	*F. oxysporum* L.	Cultured	France	[111]
*α*-*D*-Mannopyranosyl-(1→X)-inositol (**178**)	342	C_12_H_22_O_11_	*F. oxysporum* L.	Cultured	France	[111]
**Miscellaneous**						
Moniliformin (**179**)	97	C_4_HNaO_3_	*F. oxysporum* 598	*Baccharis* spp. (Asteraceae)	Brazil	[105]
			*F. oxysporum*	*Asparagus officinalis* (Asparagaceae)	Western Poland	[57]
7-Methyl-1,3,5-cyclooctatriene (MCOT) (**180**)	120	C_9_H_12_	*F. oxysporum* 13-8	*Prunus dulcis*, hull (Rosaceae)	California, USA	[109]

**Table 2:** Biological activities of the reported metabolites from *F. oxysporum*.

Compound Name	Biological Activity	Assay, Organism, or Cell Line	Biological Results	Positive Control	Ref.
Oxysporidinone (**53**)	Antifungal	Agar diffusion/*Aspergillus* niger	10 µg/mL (MIC)	-	[62]
		Agar diffusion/*Botrytis* cinerea	1 µg/mL (MIC)	-	[62]
		Agar diffusion/*Alternaria alternata*	50 µg/mL (MIC)	-	[62]
		Agar diffusion/*Venturia inequalis*	10 µg/mL (MIC)	-	[62]
(−)-Oxysporidinone (**54**)	Antifungal	MABA/*Cryptococcus neoformans*	35.0 µg/mL (IC_50_)	Amphotericin B 0.45 µg/mL (IC_50_)	[63]
		MABA/*Aspergillus fumigatus*	2.0 µg/mL (IC_50_)	Amphotericin B 0.91 µg/mL (IC_50_)	[63]
(−)-4,6′-Anhydrooxysporidinone (**57**)	Antibacterial	Microtiter plate/*B*. *subtilis*	25.0 μg/mL (MIC)	-	[65]
		Microtiter plate/MRSA	100.0 μg/mL (MIC)	-	[65]
6-*Epi*-oxysporidinone (**59**)	Antifungal	MABA/*Aspergillus fumigatus*	35.0 µg/mL (IC_50_)	Amphotericin B 0.91 µg/mL (IC_50_)	[63]
Fusaric acid (**63**)	Antimicrobial	Microtitre plate/*Colletotrichum coccodes*	81.0 µg/mL (IC_50_)	DMSO 1%	[66]
		Microtitre plate/*Magnaporthe grisea*	50.0 µg/mL (IC_50_)	DMSO 1%	[66]
		Microtitre plate/*Phytophthora capsici*	0.36 µg/mL (IC_50_)	DMSO 1%	[66]
		Microtitre plate/*Phytophthora infestans*	1.0 µg/mL (IC_50_)	DMSO 1%	[66]
		Microtitre plate/*Rhizoctonia solani*	11.0 µg/mL (IC_50_)	DMSO 1%	[66]
		Microtitre plate/*Acidovorax konjaci*	0.2 µg/mL (IC_50_)	DMSO 1%	[66]
		Microtitre plate/*Agrobacterium tumefaciens*	3.0 µg/mL (IC_50_)	DMSO 1%	[66]
		Microtitre plate/*Burkholderia glumae*	1.7 µg/mL (IC_50_)	DMSO 1%	[66]
		Microtitre plate/*Pectobacterium carotovora* ssp. *carotovora*	12.0 µg/mL (IC_50_)	DMSO 1%	[66]
		Microtitre plate/*Pseudomonas syringae* pv. *lachrymans*	11.0 µg/mL (IC_50_)	DMSO 1%	[66]
		Microtitre plate/*Xanthomonas euvesicatoria*	0.2 µg/mL (IC_50_)	DMSO 1%	[66]
Fusaricate B (**66**)	Cytotoxicity	MTT/L5178Y	37.7 µM (IC_50_)	Kahalalide F 4.3 µM (IC_50_)	[69]
Oxysporizoline (**73**)	Antioxidant	DPPH	10 μM (IC_50_)	Ascorbic acid 20 μM (IC_50_)	[70]
Butenolide (**75**)	Antioxidant	DPPH	12 μM (IC_50_)	Ascorbic acid 20 μM (IC_50_)	[70]
2-Oxo-8-azatricyclo[9.3.1.1^3,7^]-hexadeca -1(15),3(16),4,6,11,13-hexaen-10-one (**80**)	Antimicrobial	Double microdilution/*Escherichia coli* ATCC25922	1.6 µg/mL (MIC)	Ampicillin 10 µg/mL (MIC)	[73]
		Double microdilution/*Yersinia pseudotuberculosis* ATCC911	1.6 µg/mL (MIC)	Ampicillin 18 µg/mL (MIC)	[73]
		Double microdilution/*Klebsiella pneumonia* subsp. *pneumonia* ATCC13883	1.6 µg/mL (MIC)	Ampicillin 18 µg/mL (MIC)	[73]
		Double microdilution/*Pseudomonas* *aeruginosa* ATCC27853	1.6 µg/mL (MIC)	Ampicillin >128 µg/mL (MIC)	[73]
		Double microdilution/*Staphylococcus aureus* ATCC25923	0.8 µg/mL (MIC)	Ampicillin 10 µg/mL (MIC)	[73]
		Double microdilution/*Enterococcus faecalis* ATCC29212	1.6 µg/mL (MIC)	Ampicillin 35 µg/mL (MIC)	[73]
		Double microdilution/*Streptococcus mutans* RSKK07038	6.3 µg/mL (MIC)	-	[73]
		Double microdilution/*Lactobacillus casei* RSK591	12.5 µg/mL (MIC)	-	[73]
		Double microdilution/*Bacillus cereus* 702 Roma	0.8 µg/mL (MIC)	Ampicillin 15 µg/mL (MIC)	[73]
		Double microdilution/*Mycobacterium* *smegmatis* ATCC607	3.1 µg/mL (MIC)	Streptomycin 4 µg/mL (MIC)	[73]
		Double microdilution/*Candida albicans* ATCC60193	3.1 µg/mL (MIC)	Fluconazole < 8 µg/mL (MIC)	[73]
		Double microdilution/*C. tropicalis* ATTCC 13803	3.1 µg/mL (MIC)	Fluconazole < 8 µg/mL (MIC)	[73]
		Double microdilution/*Saccharomyces cerevisiae* RSKK251	6.3 µg/mL (MIC)	Fluconazole < 8 µg/mL (MIC)	[73]
Fusarioxazin (**81**)	Cytotoxicity	MTT/MCF-7	2.1 µM (IC_50_)	Doxorubicin 0.68 µM (IC_50_)	[18]
		MTT/HCT116	4.7 µM (IC_50_)	Doxorubicin 1.34 µM (IC_50_)	[18]
		MTT/A549	3.2 µM (IC_50_)	Doxorubicin 0.39 µM (IC_50_)	[18]
	Antimicrobial	Disc diffusion/*S. aureus*	5.3 µg/mL (MIC)	Ciprofloxacin 3.9 µg/mL (MIC)	[18]
		Disc diffusion/*B. cereus*	3.7 µg/mL (MIC)	Ciprofloxacin 2.3 µg/mL (MIC)	[18]
		Disc diffusion/*E. coli*	15.9 µg/mL (MIC)	Ciprofloxacin 4.1 µg/mL (MIC)	[18]
		Disc diffusion/*C. albicans*	18.2 µg/mL (MIC)	Clotrimazole 3.5 µg/mL (MIC)	[18]
*Epi*-trichosetin (**82**)	Inhibition of UPP synthase	Enzyme-coupled fluorescent/*S. aureus*	83.0 µM (IC_50_)	-	[74]
	Antimicrobial	Disc diffusion/*S. aureus* ATCC 6538P (MSSA)	18.0 mm (IZD)	-	[74]
		Disc diffusion/*S. aureus* K24 (MRSA)	16.0 mm (IZD)	-	[74]
		Disc diffusion/*Bacillus subtilis* PCI 219	18.0 mm (IZD)	-	[74]
		Disc diffusion/*Micrococcus luteus* PCI 9341	17.0 mm (IZD)	-	[74]
		Disc diffusion/*Mycobacterium smegmatis* ATCC 607	9.0 mm (IZD)	-	[74]
		Disc diffusion/*Escherichia coli* NIHJ	10.0 mm (IZD)	-	[74]
		Disc diffusion/*Xanthomonas campestris* KB 88	9.0 mm (IZD)	-	[74]
		Disc diffusion/*Bacteroides fragilis* ATCC 23745	11.0 mm (IZD)	-	[74]
		Disc diffusion/*Acholeplasma laidlawii* KB 174	12.0 mm (IZD)	-	[74]
		Disc diffusion/*Pyricularia oryzae* KF 180	14.0 mm (IZD)	-	[74]
		Disc diffusion/*Mucor racemosus* IFO 4581	7.0 mm (IZD)	-	[74]
Trichosetin (**83**)	Inhibition of UPP synthase	Enzyme-coupled fluorescent/*S. aureus*	30.0 µM (IC_50_)	-	[74]
	Antimicrobial	Disc diffusion/*S. aureus* ATCC 6538P (MSSA)	15.0 mm (IZD)	-	[74]
		Disc diffusion/*S. aureus* K24 (MRSA)	14.0 mm (IZD)	-	[74]
		Disc diffusion/*Bacillus subtilis* PCI 219	16.0 mm (IZD)	-	[74]
		Disc diffusion/*Micrococcus luteus* PCI 9341	15.0 mm (IZD)	-	[74]
		Disc diffusion/*Mycobacterium smegmatis* ATCC 607	8.0 mm (IZD)	-	[74]
		Disc diffusion/*Escherichia coli* NIHJ	10.0 mm (IZD)	-	[74]
		Disc diffusion/*Xanthomonas campestris* KB 88	7.0 mm (IZD)	-	[74]
		Disc diffusion/*Bacteroides fragilis* ATCC 23745	11.0 mm (IZD)	-	[74]
		Disc diffusion/*Acholeplasma laidlawii* KB 174	9.0 mm (IZD)	-	[74]
		Disc diffusion/*Pyricularia oryzae* KF 180	11.0 mm (IZD)	-	[74]
		Disc diffusion/*Mucor racemosus* IFO 4581	7.0 mm (IZD)	-	[74]
Enniatin A (**91**)	Antifungal	MABA/*Candida albicans*	2.0 µg/mL (IC_50_)	Amphotericin B 0.35 µg/mL (IC_50_)	[63]
		MABA/*Cryptococcus neoformans*	3.5 µg/mL (IC_50_)	Amphotericin B 0.45 µg/mL (IC_50_)	[63]
		MABA/*Mycobacterium intracellulare*	5.0 µg/mL (IC_50_)	Ciprofloxacin 0.3 µg/mL (IC_50_)	[63]
Enniatin A1 (**92**)	Antifungal	MABA/*Candida albicans*	2.0 µg/mL (IC_50_)	Amphotericin B 0.35 µg/mL (IC_50_)	[63]
		MABA/*Cryptococcus neoformans*	4.5 µg/mL (IC_50_)	Amphotericin B 0.45 µg/mL (IC_50_)	[63]
		MABA/*Mycobacterium intracellulare*	9.0 µg/mL (IC_50_)	Ciprofloxacin 0.3 µg/mL (IC_50_)	[63]
Enniatin B1 (**93**)	Antifungal	MABA/*Candida albicans*	2.0 µg/mL (IC_50_)	Amphotericin B 0.35 µg/mL (IC_50_)	[63]
		MABA/*Cryptococcus neoformans*	9.0 µg/mL (IC_50_)	Amphotericin B 0.45 µg/mL (IC_50_)	[63]
		MABA/*Mycobacterium intracellulare*	15.0 µg/mL (IC_50_)	Ciprofloxacin 0.3 µg/mL (IC_50_)	[63]
Enniatin H (**96**)	Cytotoxicity	SRB/A549	1.84 µM (EC_50_)	Doxorubicin 0.03 µM (EC_50_)	[83]
		SRB/SK-OV-3	1.71 µM (EC_50_)	Doxorubicin 0.06 µM (EC_50_)	[83]
		SRB/SK-MEL-2	1.77 µM (EC_50_)	Doxorubicin 0.04 µM (EC_50_)	[83]
		SRB/HCT15	2.45 µM (EC_50_)	Doxorubicin 0.2 µM (EC_50_)	[83]
Enniatin I (**97**)	Cytotoxicity	SRB/A549	0.50 µM (EC_50_)	Doxorubicin 0.03 µM (EC_50_)	[83]
		SRB/SK-OV-3	0.49 µM (EC_50_)	Doxorubicin 0.06 µM (EC_50_)	[83]
		SRB/SK-MEL-2	0.53 µM (EC_50_)	Doxorubicin 0.04 µM (EC_50_)	[83]
		SRB/HCT15	0.53 µM (EC_50_)	Doxorubicin 0.2 µM (EC_50_)	[83]
Enniatin MK1688 (**98**)	Cytotoxicity	SRB/A549	0.45 µM (EC_50_)	Doxorubicin 0.03 µM (EC_50_)	[83]
		SRB/SK-OV-3	0.45 µM (EC_50_)	Doxorubicin 0.06 µM (EC_50_)	[83]
		SRB/SK-MEL-2	0.63 µM (EC_50_)	Doxorubicin 0.04 µM (EC_50_)	[83]
		SRB/HCT15	0.53 µM (EC_50_)	Doxorubicin 0.2 µM (EC_50_)	[83]
Beauvericin (**99**)	Cytotoxicity	MTT/NCI-H460	0.43 µM (IC_50_)	Doxorubicin 0.01 µM (IC_50_)	[64]
		MTT/MIA Pa Ca-2	0.26 µM (IC_50_)	Doxorubicin 0.05 µM (IC_50_)	[64]
		MTT/MCF-7	0.42 µM (IC_50_)	Doxorubicin 0.07 µM (IC_50_)	[64]
		MTT/SF-268	0.38 µM (IC_50_)	Doxorubicin 0.04 µM (IC_50_)	[64]
		SRB/A549	1.43 µM (EC_50_)	Doxorubicin 0.03 µM (EC_50_)	[83]
		SRB/SK-OV-3	1.39 µM (EC_50_)	Doxorubicin 0.06 µM (EC_50_)	[83]
		SRB/SK-MEL-2	1.47 µM (EC_50_)	Doxorubicin 0.04 µM (EC_50_)	[83]
		SRB/HCT15	1.86 µM (EC_50_)	Doxorubicin 0.2 µM (EC_50_)	[83]
		MTT/PC-3	49.5 µM (IC_50_)	Cisplatin 26.8 µM (IC_50_)	[65]
		MTT/ PANC-1	47.2 µM (IC_50_)	Cisplatin 26.2 µM (IC_50_)	[65]
		MTT/A549	10.4 µM (IC_50_)	Cisplatin 19.8 µM (IC_50_)	[65]
		MTT/HCT-8	3.02 µg/mL (IC_50_)	Doxorubicin 0.04 µg/mL (IC_50_)	[84]
		MTT/MDA-MB435	3.17 µg/mL (IC_50_)	Doxorubicin 0.2 µg/mL (IC_50_)	[84]
		MTT/SF295	2.39 µg/mL (IC_50_)	Doxorubicin 0.04 µg/mL (IC_50_)	[84]
	Antibacterial	Microtiter plate/MRSA	3.125 μg/mL (MIC)	-	[65]
		Microtiter plate/*B*. *subtilis*	3.125 μg/mL (MIC)	-	[65]
	Leishmanicidal	MTT (anti-promastigote)/*L*. *braziliensis* strain H3227 (MHOM/BR/94/H-3227)	1.86 μM (EC_50_)	Geneticin 0.007 HOM/EC_50_)	[84]
Anhydrofusarubin (**105**)	Cytotoxicity	MTT/HCT-8	9.85 µg/mL (IC_50_)	Doxorubicin 0.04 µg/mL (IC_50_)	[84]
		MTT/MDA-MB435	6.23 µg/mL (IC_50_)	Doxorubicin 0.2 µg/mL (IC_50_)	[84]
		MTT/SF295	6.32 µg/mL (IC_50_)	Doxorubicin 0.04 µg/mL (IC_50_)	[84]
	Plant nematicidal	Antinemic/*Meloidogyne incognita*	257.6 µg/mL (LC_50_)	Carbofuran 54.2 µg/mL (LC_50_)	[88]
		Antinemic/*Rotylenchulus reniformis*	285.3 µg/mL (LC_50_)	Carbofuran 37.6 µg/mL (LC_50_)	[88]
Fusarubin (**107**)	Plant nematicidal	Antinemic/*Meloidogyne incognita*	248.9 µg/mL (LC_50_)	Carbofuran 54.2 µg/mL (LC_50_)	[88]
		Antinemic/*Rotylenchulus reniformis*	301.6 µg/mL (LC_50_)	Carbofuran 37.6 µg/mL (LC_50_)	[88]
8-*O*-methylfusarubin (**108**) = 9-*O*-Methylfusarubin	Plant nematicidal	Antinemic/*Meloidogyne incognita*	376.4 µg/mL (LC_50_)	Carbofuran 54.2 µg/mL (LC_50_)	[88]
		Antinemic/*Rotylenchulus reniformis*	518.4 µg/mL (LC_50_)	Carbofuran 37.6 µg/mL (LC_50_)	[88]
3-*O*-Methyl-8-*O*-methylfusarubin (**109**) = 3-*O*-Methyl-9-*O*-methylfusarubin	Plant nematicidal	Antinemic/*Meloidogyne incognita*	478.5 µg/mL (LC_50_)	Carbofuran 54.2 µg/mL (LC_50_)	[88]
		Antinemic/*Rotylenchulus reniformis*	465.2 µg/mL (LC_50_)	Carbofuran 37.6 µg/mL (LC_50_)	[88]
8-*O*-Methyljavanicin (**111**)	Antibacterial	Microdilution/*S. aureus*	4 µg/mL (MIC)	-	[90]
		Microdilution/*Streptococcus* *pyogenes*	>128 µg/mL (MIC)	-	[90]
8-*O*-Methylsolaniol (**112**)	Antibacterial	Microdilution/*S. aureus*	32.0 µg/mL (MIC)	-	[90]
		Microdilution/*Streptococcus* *pyogenes*	64.0 µg/mL (MIC)	-	[90]
Nectriafurone (**114**)	Antibacterial	Microdilution/*S. aureus*	>128 µg/mL (MIC)	-	[90]
		Microdilution/*Streptococcus* *pyogenes*	128 µg/mL (MIC)	-	[90]
Bikaverin (**119**)	Antimicrobial	Microtitre plate/*Colletotrichum coccodes*	70.0 µg/mL (IC_50_)	DMSO 1%	[66]
		Microtitre plate/*Magnaporthe grisea*	70.0 µg/mL (IC_50_)	DMSO 1%	[66]
		Microtitre plate/*Phytophthora capsici*	10.0 µg/mL (IC_50_)	DMSO 1%	[66]
		Microtitre plate/*Phytophthora infestans*	60.0 µg/mL (IC_50_)	DMSO 1%	[66]
	Cytotoxicity	MTT/NCI-H460	1.41 µM (IC_50_)	Doxorubicin 0.01 µM (IC_50_)	[64]
		MTT/MIA Pa Ca-2	1.66 µM (IC_50_)	Doxorubicin 0.05	[64]
		MTT/MCF-7	1.81 µM (IC_50_)	Doxorubicin 0.07 µM (IC_50_)	[64]
		MTT/SF-268	2.29 µM (IC_50_)	Doxorubicin 0.04 µM (IC_50_)	[64]
	Nematicidal	Antinemic/*Meloidogyne incognita*	392.9 µg/mL (LC_50_)	Carbofuran 54.2 µg/mL (LC_50_)	[88]
		Antinemic/*Rotylenchulus reniformis*	618.0 µg/mL (LC_50_)	Carbofuran 37.6 µg/mL (LC_50_)	[88]
Wortmannin (**129**)	Antifungal	MABA/*Candida albicans*	0.25 µg/mL (IC_50_)	Amphotericin B 0.35 µg/mL (IC_50_)	[63]
H1-A = Ergosta-5,8 (14),22-trien-7-one, 3-hydroxy-(3*β*, 22E) (**130**)	Anti-hepatitis C virus (HCV) NS3 serine protease	FRET/HCV NS3 protease	99.7 μM/L (Ki value)	VX950 3.5 μM/L (Ki value)	[99]
Fusariumins C (**135**)	Antimicrobial	Microbroth dilution/*S. aureus*	6.25 μM (MIC)	Ampicillinum sodium	[102]
Fusariumins D (**136**)	Antimicrobial	Microbroth dilution/*S. aureus*	25 μM (MIC)	Ampicillinum sodium	[102]
(1-Benzyl-2-methoxy-2-oxoethyl)-2-hydroxy-3-methylbutanoate (**153**)	Cytotoxicity	MTT/MCF-7	15.01 µM (IC_50_)	Doxorubicin 0.053 µM (IC_50_)	[73]
		MTT/PC-3	19.13 µM (IC_50_)	Doxorubicin 0.09 µM (IC_50_)	[73]
		MTT/A549	17.06 µM (IC_50_)	Doxorubicin 17.75 µM (IC_50_)	[73]
	Antimicrobial	Double microdilution/*Escherichia coli* ATCC25922	60 µg/mL (MIC)	Ampicillin 10 µg/mL (MIC)	[73]
		Double microdilution/*Yersinia pseudotuberculosis* ATCC911	60 µg/mL (MIC)	Ampicillin 18 µg/mL (MIC)	[73]
		Double microdilution/*Klebsiella pneumonia* subsp. *pneumonia* ATCC13883	60 µg/mL (MIC)	Ampicillin 18 µg/mL (MIC)	[73]
		Double microdilution/*Pseudomonas aeruginosa* ATCC27853	60 µg/mL (MIC)	Ampicillin >128 µg/mL (MIC)	[73]
		Double microdilution/*Staphylococcus aureus* ATCC25923	0.94 µg/mL (MIC)	Ampicillin 10 µg/mL (MIC)	[73]
		Double microdilution/*Enterococcus faecalis* ATCC29212	1.8 µg/mL (MIC)	Ampicillin 35 µg/mL (MIC)	[73]
		Double microdilution/*Streptococcus mutans* RSKK07038	1.8 µg/mL (MIC)	-	[73]
		Double microdilution/*Lactobacillus casei* RSK591	30 µg/mL (MIC)	-	[73]
		Double microdilution/*Bacillus cereus* 702 Roma	0.94 µg/mL (MIC)	Ampicillin 15 µg/mL (MIC)	[73]
		Double microdilution/*Mycobacterium* *smegmatis* ATCC607	0.47 µg/mL (MIC)	Streptomycin 4 µg/mL (MIC)	[73]
		Double microdilution/*Candida albicans* ATCC60193	60 µg/mL (MIC)	Fluconazole < 8 µg/mL (MIC)	[73]
		Double microdilution/*C. tropicalis* ATTCC 13803	60 µg/mL (MIC)	Fluconazole < 8 µg/mL (MIC)	[73]
		Double microdilution/*Saccharomyces cerevisiae* RSKK251	120 µg/mL (MIC)	Fluconazole < 8 µg/mL (MIC)	[73]
Mixture of 2,3-dihydroxypropanoic (11*Z*)-octadecenoic anhydride (**168**) and 2,3-dihydroxypropanoic, (9*E*,12*E*)-octadecadienoic anhydride (**169**)	Antimicrobial	Double microdilution/*E. coli* ATCC25922	7.8 µg/mL (MIC)	Ampicillin 10 µg/mL (MIC)	[73]
		Double microdilution/*Yersinia* *pseudotuberculosis* ATCC911	7.7 µg/mL (MIC)	Ampicillin 18 µg/mL (MIC)	[73]
		Double microdilution/*Klebsiella pneumonia* subsp. *pneumonia* ATCC13883	7.7 µg/mL (MIC)	Ampicillin 18 µg/mL (MIC)	[73]
		Double microdilution/*Pseudomonas* *aeruginosa* ATCC27853	7.7 µg/mL (MIC)	Ampicillin > 128 µg/mL (MIC)	[73]
		Double microdilution/*Staphylococcus aureus* ATCC25923	3.8 µg/mL (MIC)	Ampicillin 10 µg/mL (MIC)	[73]
		Double microdilution/*Enterococcus faecalis* ATCC29212	7.7 µg/mL (MIC)	Ampicillin 35 µg/mL (MIC)	[73]
		Double microdilution/*Streptococcus mutans* RSKK07038	30.6 µg/mL (MIC)	-	[73]
		Double microdilution/*Lactobacillus casei* RSK591	61.2 µg/mL (MIC)	-	[73]
		Double microdilution/*Bacillus cereus* 702 Roma	7.7 µg/mL (MIC)	Ampicillin 15 µg/mL (MIC)	[73]
		Double microdilution/*Mycobacterium* *smegmatis* ATCC607	30.6 µg/mL (MIC)	Streptomycin 4 µg/mL (MIC)	[73]
		Double microdilution/*C. albicans* ATCC60193	30.6 µg/mL (MIC)	Fluconazole < 8 µg/mL (MIC)	[73]
		Double microdilution/*C. tropicalis* ATTCC 13803	30.6 µg/mL (MIC)	Fluconazole < 8 µg/mL (MIC)	[73]
		Double microdilution/*Saccharomyces cerevisiae* RSKK251	30.6 µg/mL (MIC)	Fluconazole < 8 µg/mL (MIC)	[73]
Mixture of 3-hydroxy-1,2,6,10-tetramethylundecyl hexzadecanoate (**170**), 3-hydroxy-1,2,6,10-tetramethylundecyl (9E)-octadecaenoate (**171**), and 3-hydroxy-1,2,6,10-tetramethylundecyl octadecanoate (**172**)	Cytotoxicity	MTT/MCF-7	7.75 µM (IC_50_)	Doxorubicin 0.053 µM (IC_50_)	[73]
		MTT/PC-3	17.75 µM (IC_50_)	Doxorubicin 0.09 µM (IC_50_)	[73]
		MTT/A549	7.51 µM (IC_50_)	Doxorubicin 17.75 µM (IC_50_)	[73]
	Antimicrobial	Double microdilution/*S. aureus* ATCC25923	4.3 µg/mL (MIC)	Ampicillin 10 µg/mL (MIC)	[73]
		Double microdilution/*Enterococcus faecalis* ATCC29212	4.3 µg/mL (MIC)	Ampicillin 35 µg/mL (MIC)	[73]
		Double microdilution/*Streptococcus mutans* RSKK07038	4.3 µg/mL (MIC)	-	[73]
		Double microdilution/*Bacillus cereus* 702 Roma	2.1 µg/mL (MIC)	Ampicillin 15 µg/mL (MIC)	[73]

## Data Availability

Not applicable.

## Abbreviations

A549	Human non-small-cell lung cancer cell line
AgNPs	Silver nanoparticles; AGS: Gastric adenocarcinoma
APase	β-L-Arabinopyranosidase
BGL	β-Glucosidases
Bi_2_S_3_	Bismuth sulphide
C26	Murine colon carcinoma
CBP	Consolidated bioprocessing
CdSe	Cadmium/selenium
CLEAs	Cross-linked enzyme aggregates
CLSM	Confocal laser scanning microscopic
DHAP	Dihydroxyactetone-3-phosphate
DHBA	2,3-Dihydroxybenzoic acid
DPPH	2,2-Diphenyl-1-picrylhydrazyl
EC_50_	Concentration required inhibiting cell growth in vitro by 50%
ESBL	Extended-spectrum beta-lactamase
FAOD	Fructosyl amino acid oxidase
FLC	Fluconazole
FLO	Fructosyl lysine oxidase
FRET	Fluorescence resonance energy transfer
FUC	α-L-Fucosidase
GAP	Glyceraldehyde-3-phosphate
GH-11	Glycosyl hydrolase family 11
GNPs	Gold nanoparticles
GPase	α-D-Galactopyranosidase
HaCaT	Human immortalized keratinocyte cells
HCT116	Human colorectal adenocarcinoma
HCT15	Human colorectal cancer cell line
HCT8	Human colon tumor cell lines
HeLa	Human cervix carcinoma cell line
HepG2	Human liver cancer cell line
HUVEC-2	Human umbilical vascular endothelial cells
IC_50_	Concentration causing 50% growth inhibition
IL	Ionic liquid
IZD	Inhibition zone diameter
L5178Y	Mouse lymphoma cell line
LC	Lethal concentration
LDH	Lactate dehydrogenase release assay
LMS	Laccase mediator system
LOX	Lipoxygenase
MABA	Microplate Alamar blue assay
MB	Methylene blue
MCF-7	Breast cancer cell line
MDA-MB-231	Metastatic breast cancer cell line
MDA-MB435	Human melanoma tumor cell lines
MDR	Multidrug-resistant
MIA Pa Ca-2	Pancreati cancer cell line
MIC	Minimum inhibitory concentration
MRSA	Methicillin-resistant *Staphylococcus aureus*
MRSA	Methicillin-resistant *S*. *aureus*
MTT	(3-(4,5-Dimethylthiazol-2-yl)-2,5-diphenyltetrazolium bromide)
N_2_O	Dinitrogenoxide
NCI-H460	Non-small-cell lung
NO	Nitrogen monoxide
NOR	Nitric oxide reductase
NPs	Nanoparticles
OMW	Olive Mill Waste
PANC-1	Human Pancreatic cancer cell line
PC-3	Human prostate carcinoma cell line
PC-3M	Metastatic prostate cancer cell line
PDA	Potato dextrose agar
PET	Polyethylene terephthalate
PLB	Phospholipase B
PNP-α-D-Galp	*Para*-nitrophenyl α-D-galactopyranoside
PNP-β-L-Arap	*Para*-nitrophenyl α-l-arabinopyranoside
SF-268	Central nervous system glioma
SF295	Human brain tumor cell lines
SK-MEL-2	Skin cancer cell line
SK-OV-3	Ovarian cancer cell line
SRB	Sulforhodamine B
TAG	Triacylglycerols
TPI	Triosephosphate isomerase
UPP	Undecaprenyl pyrophosphate
VOCs	Volatile organic compounds
WHA	Wound healing assay
